# An automatic sustained attention prediction (ASAP) method for infants and toddlers using wearable device signals

**DOI:** 10.1038/s41598-025-96794-x

**Published:** 2025-04-17

**Authors:** Yisi Zhang, A. Priscilla Martinez-Cedillo, Harry T. Mason, Quoc C. Vuong, M. Carmen Garcia-de-Soria, David Mullineaux, Marina I. Knight, Elena Geangu

**Affiliations:** 1 Department of Psychological and Cognitive Sciences, Tsinghua University, Beijing, 100084 People’s Republic of China; 2 Department of Psychology, University of York, York, YO10 5DD England; 3 Department of Psychology, University of Essex, Wivenhoe Park, Colchester, Essex, CO4 3SQ England; 4 School of Physics, Engineering and Technology, University of York, York, YO10 5DD England; 5 Bristol Medical School, University of Bristol, Oakfield House, Bristol, BS8 2BN England; 6 Bioscience Institute, Newcastle University, Newcastle Upon Tyne, NE1 7RU England; 7 School of Psychology, Newcastle University, Newcastle Upon Tyne, NE1 7RU England; 8 Department of Psychology, University of Aberdeen, Aberdeen, UK; 9 Department of Mathematics, University of York, York, YO10 5DD England

**Keywords:** Sustained attention, Infant development, Computational model, Electrocardiogram, Wearable sensors, Naturalistic studies, Visual saliency, Neuroscience, Mathematics and computing

## Abstract

Sustained attention (SA) is a critical cognitive ability that emerges in infancy and affects various aspects of development. Research on SA typically occurs in lab settings, which may not reflect infants’ real-world experiences. Infant wearable technology can collect multimodal data in natural environments, including physiological signals for measuring SA. Here we introduce an automatic sustained attention prediction (ASAP) method that harnesses electrocardiogram (ECG) and accelerometer (Acc) signals. Data from 75 infants (6- to 36-months) were recorded during different activities, with some activities emulating those occurring in the natural environment (i.e., free play). Human coders annotated the ECG data for SA periods validated by fixation data. ASAP was trained on temporal and spectral features from the ECG and Acc signals to detect SA, performing consistently across age groups. To demonstrate ASAP’s applicability, we investigated the relationship between SA and perceptual features—saliency and clutter—measured from egocentric free-play videos. Results showed that saliency in infants’ and toddlers’ views increased during attention periods and decreased with age for attention but not inattention. We observed no differences between ASAP attention detection and human-coded SA periods, demonstrating that ASAP effectively detects SA in infants during free play. Coupled with wearable sensors, ASAP provides unprecedented opportunities for studying infant development in real-world settings.

## Introduction

Sustained attention (SA) is a fundamental cognitive ability that emerges during infancy and has a widespread impact on various developmental domains^1–3^. As a form of endogenous attention, SA refers to the ability to focus on a particular stimulus or event over an extended period, even with distractors present^4^. SA shapes infant learning^5–7^ and is associated with the emergence in infancy and early childhood of more complex cognitive and social abilities, such as executive function^5,8^, self-regulation^4,9–12^, memory^1,13,14^, language, and social communication^15,16^. However, most developmental SA research relies on lab-based paradigms that fail to capture the complexity of infants’ day-to-day environment^7,9,17^. Infants’ everyday experiences, including the seamless flow of events and social interactions, can vary enormously both within and across cultures^18–22^. By studying these free-flowing events, we can understand the impact of the stimuli-rich nature of real-life situations on infants’ attentional processes, as well as the similarities and differences with the paradigms implemented in the laboratory^23–25^. It is also possible that phenomena otherwise not observed or studied in the laboratory may occur in the real-world contexts, and systematic observations based on multimodal data may lead to their discovery.

The development of affordable, lightweight and user-friendly wearable technology for infants has facilitated the recording of what infants see and hear in natural settings, and related autonomic nervous system (ANS) responses^26^. These recordings have great potential for precise measurement and characterisation of SA development as a function of mutual interactions with various factors^27^. However, a key challenge in adopting ecologically valid approaches is the lack of efficient methods for extracting reliable measures of SA from the available modalities and the vast amount of data^28^. In this study, we reduce this bottleneck by introducing an innovative automatic attention detection algorithm that harnesses infant electrocardiography (ECG) and accelerometer (Acc) signals recorded with wearable sensors.

Traditionally, looking times have been the predominant measure for visual attention in infancy^29–31^. Trained researchers can determine whether infants are looking either towards or away from stimuli in various set-ups (e.g., computer-based presentations; social interactions), provided there is an accurate view of the infant’s eyes relative to the stimuli of interest^32^. Due to this approach’s simplicity and the relatively low cost of the necessary technology, looking times have been widely adopted in infant development research, enabling the investigation of many important questions. Consequently, it is unsurprising that, in recent years, algorithms have been developed to automate and enhance this approach^32^.

However, evidence from brain imaging and psychophysiology shows that not all infant looking times reflect active cognitive processing. Specifically, continuous periods of looking estimated by trained researchers can encompass both visual SA and other attentional processes, and inattention^14,33,34^. In contrast, heart rate (HR) changes (deceleration and acceleration) have been shown to more effectively differentiate between attentional processes, particularly for visual SA^7,14,35^, and inattention. Orienting towards a stimulus of interest is typically characterized by rapid HR deceleration relative to baseline or pre-stimulus levels. If attention orientation is followed by SA for further processing, HR stabilises at the lower rate for 2–20 s, but can be even longer^35^. This reduced HR is often also accompanied by decreased body movement^36^. Attention termination (disengagement from processing the stimulus of interest) is marked by a rapid HR acceleration to approximate pre-attention baseline levels^5^. HR-defined measures of infant SA (HRDSA), but not generic bouts of looking have been associated with neural indicators of active attention and in-depth information processing such as frontal electroencephalogram (EEG) synchronisation in theta oscillations, an effect observed even in infants as young as 3 months^5,7^. These findings support HRDSA as a robust method for studying the development of SA in infancy.

Building on HRDSA, we developed an automatic SA prediction (ASAP) method to detect attention periods using ECG and Acc signals obtained from wearable sensors commonly used in infant research^26,37,38^. To train and validate this method, we created a ground-truth dataset consisting of ECG and Acc signals, human-coded SA annotations, and eye-tracking data collected in the laboratory which was set up to emulate home situations (e.g., play mat with toys). ASAP can thus be applied to wearable sensor data collected in natural environments, providing a non-invasive means of studying infant SA. One potential application involves combining ASAP with recordings from lightweight, wireless head-mounted cameras. These cameras, which are well tolerated by infants, can continuously capture the diversity of visual information present in their egocentric views over extended periods of time. However, on their own, these data may not clearly indicate when visual information is likely to be processed in depth. The integration of HRDSA derived from wearable body sensors synchronised with head-mounted cameras can overcome these limitations, enabling researchers to differentiate between egocentric views during which infants manifest sustained attention vs. inattention.

ASAP can be decomposed into three primary steps, motivated by the abrupt HR deceleration and acceleration that occur during attention orientation and termination, immediately before and after the period of SA. The first step involves change point detection (CPD)^39,40^, which identifies time points when the statistical properties of a signal undergo significant changes. Infancy and toddlerhood are characterised by frequent spans of SA^41,42^, suiting a CPD algorithm which can objectively identify multiple change points within HR time series data. In the second step, momentary attention detection is formulated as a binary classification task. The classification model is trained with a curated set of features using a feature selection process. In the final step, the segmentation of SA periods is refined to preserve their temporal structure.

The temporal and spectral features of HR fluctuations provide rich information about the ANS’ regulation of homeostasis, physiological arousal, and cognitive states^43,44^. Precisely, heart rate variability (HRV)—measured through variations in beat-to-beat intervals in the time domain or through low (0.04–0.15 Hz) and high-frequency (0.15–0.4 Hz) HR oscillations in the frequency domain—is associated with sympathetic and parasympathetic nervous activity^45^. Higher HRV is linked to better performance in SA tasks (e.g., continuous performance tests involving executive functions^46^). Low-frequency HR oscillations are related to attentional demands^47,48^, and an increased low-to-high frequency power ratio is associated with poor attention in children with attention deficit hyperactivity disorder (ADHD)^49^. We characterise HR fluctuations in both time and frequency domains using a wavelet transform to capture dynamic HRV changes, potentially relating to momentary shifts in attentional states. Wavelet analysis has been widely applied to characterise instantaneous features of physiological signals^50–52^. Given the tight coordination between movement and cardiac output^53^, we also include Acc signals and investigate their dynamic coupling with HR in time and frequency for additional attention insights.

To demonstrate ASAP’s utility in attention research, we apply ASAP in a study examining what drives SA when infants and toddlers actively engage with people and objects. Attention allocation can be influenced by factors such as low-level visual features (e.g., strong edges, bright colours, large movements in the scene), people (e.g., faces and bodies), and objects (e.g., toys)^29,54–64^. However, most previous studies present images or videos under constrained lab conditions (e.g., sitting on a caregiver’s lap with stimuli presented on a monitor), which fail to capture infants’ and toddlers’ active interactions with their environment, and how this may change throughout development. Here we focus on visual saliency^65^ and visual clutter^66^ extracted from observers’ egocentric views during free play. Additionally, we examine how these measures differ across fixated regions during attention and inattention periods, as determined by the ASAP method or human coders, and assess whether they vary with age.

## Methods

### Overview of approach

Our approach to developing an automatic SA-detection method hinges on two pivotal ideas: first, creating a lab-based ground-truth dataset with human-coded SA; second, training a model using this dataset to automatically detect SA based on ECG and Acc signals.

**Fig. 1:**
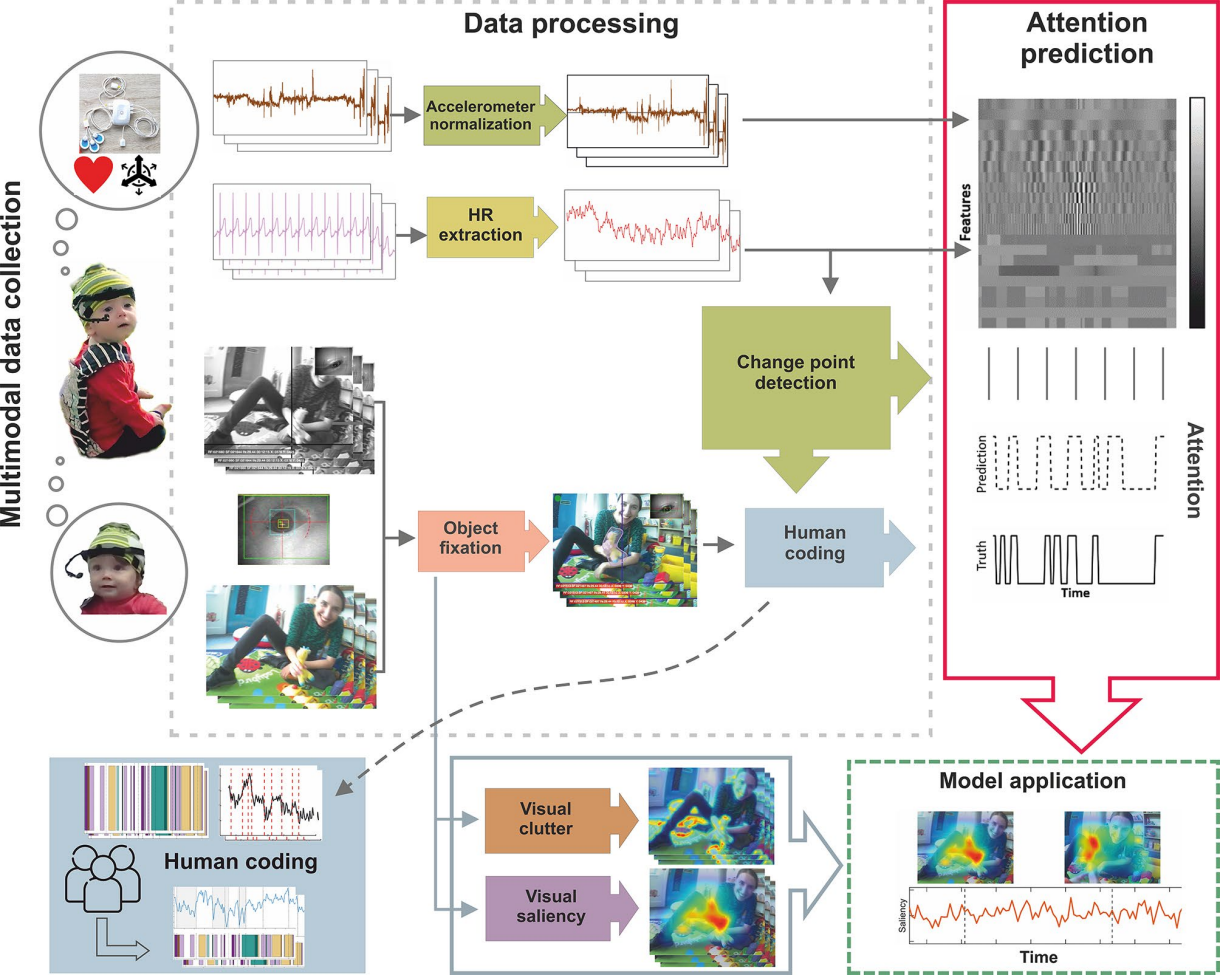
A pipeline schematic for data collection to train the SA prediction model. Two synchronised wearable devices record the data: the ECG/Acc body sensors (top cloud) and the head-mounted eye tracker (bottom cloud). During data processing, HR is extracted and then undergoes change points detection (CPD) to facilitate human coding of attention, validated by object fixation (objects coded by colours). During attention prediction, HR- and Acc-derived time series and change point segmentation form the feature matrix for training a machine learning model to predict attention periods. During model application, visual clutter and saliency signals are extracted from video frames to evaluate the model’s effectiveness.

The multimodal dataset comprises four synchronised sensor signals: ECG, Acc, the scene videos, and fixation-label data. ECG and Acc signals are simultaneously captured from a single wearable device attached to the infant’s chest. Fixation data are obtained from a head-mounted eye tracker that records infants’ and toddlers’ gaze points (Fig. 1), which determine the objects fixated upon during recording. HR is derived from the ECG signal^26,67^. We annotated attention periods synchronised with the HR and Acc signals by considering both the distinctive HR waveforms during periods of SA and human inspection of aligned fixation data (Fig. 1, “Human Coding”).

The model development uses HR and Acc signals alongside human-coded attention (Fig. 1, “Attention Prediction”) and it involves three key stages: (1) coarse segmentation of potential periods of SA based on HRDSA facilitated by CPD; (2) classification employing HR and Acc features; and (3) fragment refinement to reconstruct the temporal structure. The model’s innovation lies in several aspects: first, the integration of a CPD algorithm to objectively identify abrupt changes in HR, adapting previous lab-based procedures^35^ to naturalistic settings; second, the exploitation of temporal and spectral properties of HR and Acc signals and their interaction to delineate attentional states; and finally, preserving the temporal structure to retain the associated statistical characteristics throughout the attention detection process. We then demonstrate the model’s application in studying visual SA development by leveraging the egocentric video and fixation data collected with the HR and Acc signals (Fig. 1, “Model Application”).

### The dataset

#### Participants

In line with previous literature^68^, a sample size of *N* = 75, 6- to 36-month-old infants and toddlers were included in the final analyses (Table 1). A further 23 participants responded to the invitation to participate in the study but were excluded from the final analysis due to either refusal to wear at least one of the devices (*N* = 15) or technical errors (*N* = 8). Participants were recruited from the urban area of York, in England. Caregivers provided written informed consent before the experimental procedure began, and families received £10 and a book. For those participants depicted in figures, caregivers provided consent for publication of identifying information/images in an online open-access publication. The research presented in this empirical report received ethical approval from the Department of Psychology Ethics Committee, University of York (Identification Number – 119). The experimental procedures were conducted in adherence to the principles of the 1964 Declaration of Helsinki 8 and its later amendments.

**Table 1:** The cohort breakdown of the participants in the Dataset.

Age	N (Total = 75)	M age (months)	SD age (months)	Mean duration of recording (minutes)	SD duration (minutes)
6-months	15 (6 females)	6.43	0.48	25.09	6.81
9-months	15 (8 females)	9.17	0.47	26.27	7.58
12-months	15 (7 females)	12.47	0.99	25.78	4.62
24-months	16 (8 females)	25.50	1.98	29.99	7.55
36-months	14 (8 females)	36.16	2.86	32.51	7.22

#### Data acquisition and processing procedure

The experiment took place in a laboratory room with controlled lighting containing toys and objects, and a separate larger play area resembling a typical playroom with age-appropriate toys and books (Fig. S1). There were three tasks: check-this-out game, spin-the-pots task, and free play. Participants wore a head-mounted eye tracker to record scene video and gaze points, and body sensors to record ECG and Acc. Further details are provided in the Supplementary Information (*1. Task description*).

#### Head-mounted eye tracker data recording and processing

Eye movements were recorded using a head-mounted eye tracker (Positive Science, New York, USA), which tracked the right eye at 30 Hz. Scene recordings were captured at 30 fps with 640 × 480-pixel resolution and a wide lens (W 81.88° × H 67.78° × D 95.30°). The Yarbus software (version 2.4.3, Positive Science) was used to map participants’ gaze points onto the scene video and calibrate the eye tracker offline, accounting for variations in eye morphology and the spatial location of fixated objects^41,69^ (further details in the Supplementary Information—*2. Head-mounted eye-tracker calibration protocol*). We calculated fixations from gaze points and labelled them using the GazeTag software (version 1, Positive Science). We only include fixations with a duration > 100 ms. The labels represent 87 different items including toys, body parts, and other objects in the room. Approximately 10% of frames were unsuitable for labelling due to abrupt movements, participants removing the eye tracker, or technical errors.

#### Cardiac activity and body movement recording

**Fig. 2:**
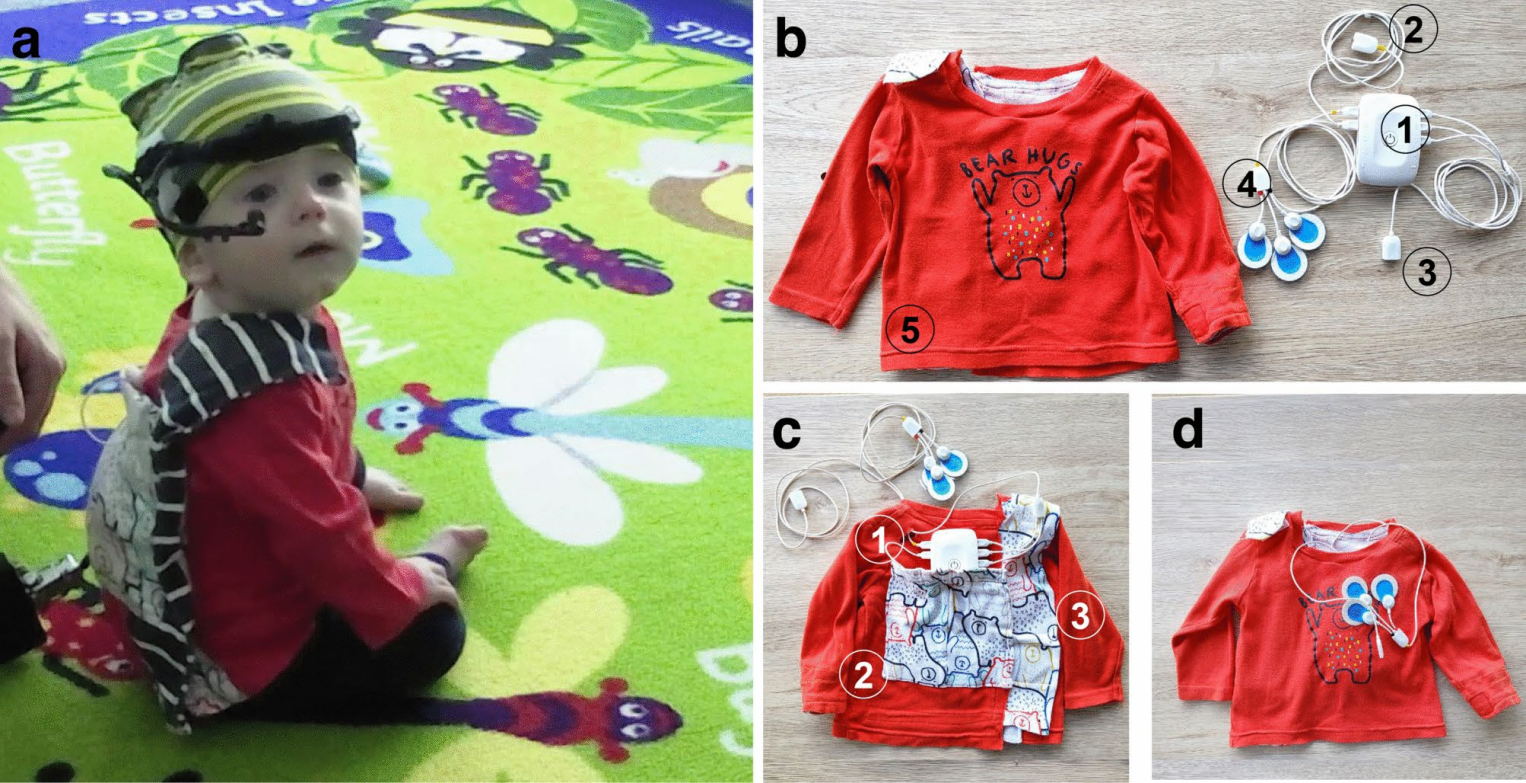
The wearable body sensor. (**a**) Example of a 9-months-old infant wearing the head-mounted eye tracker with the body sensors. (**b**) The Biosignals Plux wearable sensors: 1—the data recording hub; 2—the light sensor; 3—the acceleration sensor; 4—the ECG sensor with the Ambu blue electrodes (Ambu, Copenhagen, Denmark) attached; 5—the custom-made shirt. (**c**) The back view of the shirt showing how the sensors were embedded: 1—eyelet, which allows the ECG and Acc sensors to be brought from the back to the front and positioned on the left side of the chest; 2—the back pocket, which holds the data recording hub; 3—the shirt closes at the back via hook and loop. (**d**) Front view of the shirt with the sensors embedded, illustrating the location of the ECG sensor.

ECG and Acc signals were recorded concurrently at 500 Hz using the Biosignalsplux device (PLUX Biosignals, Lisbon, Portugal). The ECG sensor’s three-electrode montage (including ground) was attached to the left side of the chest, and the Acc sensor was placed at roughly the same location (Fig. 2). The device also includes a light sensor, enabling synchronisation of ECG/Acc data with the eye-tracking data. To ensure quick sensor placement, the Biosignalsplux hub and sensors were embedded in a custom-made shirt (Fig. 2c). Previous infant studies have also considered the movement of the head and/or limbs using various methods (e.g., behavioural videography, desk-mounted eye-tracking, wireless sensors)^53,70,71^. As the accelerometer available to us was tethered, we decided to only include the device placed on the chest. An additional head accelerometer would restrict head-movement due to additional cables and increase both the preparation time and the number of visible pieces of equipment, which could negatively impact participant compliance.

#### Sensor signal preprocessing and HR extraction

The triaxial accelerometer (Acc) data was centred by subtracting 2^15^ and then normalised by dividing by 5000. The magnitude of acceleration was calculated as the L2-norm of the triaxial acceleration.

ECG processing and R-peak detection was handled using Python^26,67^, adapted from Neurokit2 code^72^. All R-peaks were then visually inspected; mislabelled peaks were manually corrected. The HR was calculated using the time interval between consecutive R-peaks (Δt_peaks_): *HR (bpm)* = *60/Δt*_*peak*s_. The detailed procedure for noise correction has been described elsewhere^67^. The parameters used in this study are in the Supplementary Information (*3. ECG signal preprocessing and HR extraction*).

Bluetooth connectivity issues caused ECG/Acc data loss six times in total across all 75 participants, with a mean data loss duration of 159.1 s (*SD* = 106.5 s; range = 28.9–279.5 s). No HR was calculated during these periods.

#### Human coding of sustained attention

We adapted the lab-based criteria for HRDSA for data acquired under our more naturalistic conditions. In particular, we defined SA as: (Criterion 1) occurring during periods with a deceleration in participants’ HR followed later in the period by an HR acceleration^35,73–77^; and (Criterion 2) when participants fixate on and/or engage with a small number of objects during this time^78^. Detecting SA in naturalistic settings, such as free play, presents challenges compared to controlled lab conditions. Infants and toddlers in the lab have restricted movements and their HR is measured during a baseline period before an engaging stimulus is presented on a screen. However, free play lacks defined event structures and infants and toddlers can actively fixate and interact with objects, making it difficult to determine baseline HR measurements.

To address these challenges, we first developed a method to identify candidate periods corresponding to Criterion 1. We used a CPD algorithm to automatically detect abrupt decreases (SA onsets) and increases (SA terminations) in HR without the need for a pre-determined baseline (see *The Automatic Sustained Attention Prediction (ASAP) Model* section). This provides an objective approach to detect abrupt changes by adaptively responding to the data, minimising the need for subjective, user-defined criteria, and holding the potential to accommodate variations attributable to individual differences, fluctuations in HR baseline, and the developmental change of HR. Second, we use eye-tracking data to determine whether these periods were associated with infants fixating on or following objects within the scene corresponding to Criterion 2.

#### Human coding protocol

**Fig. 3:**
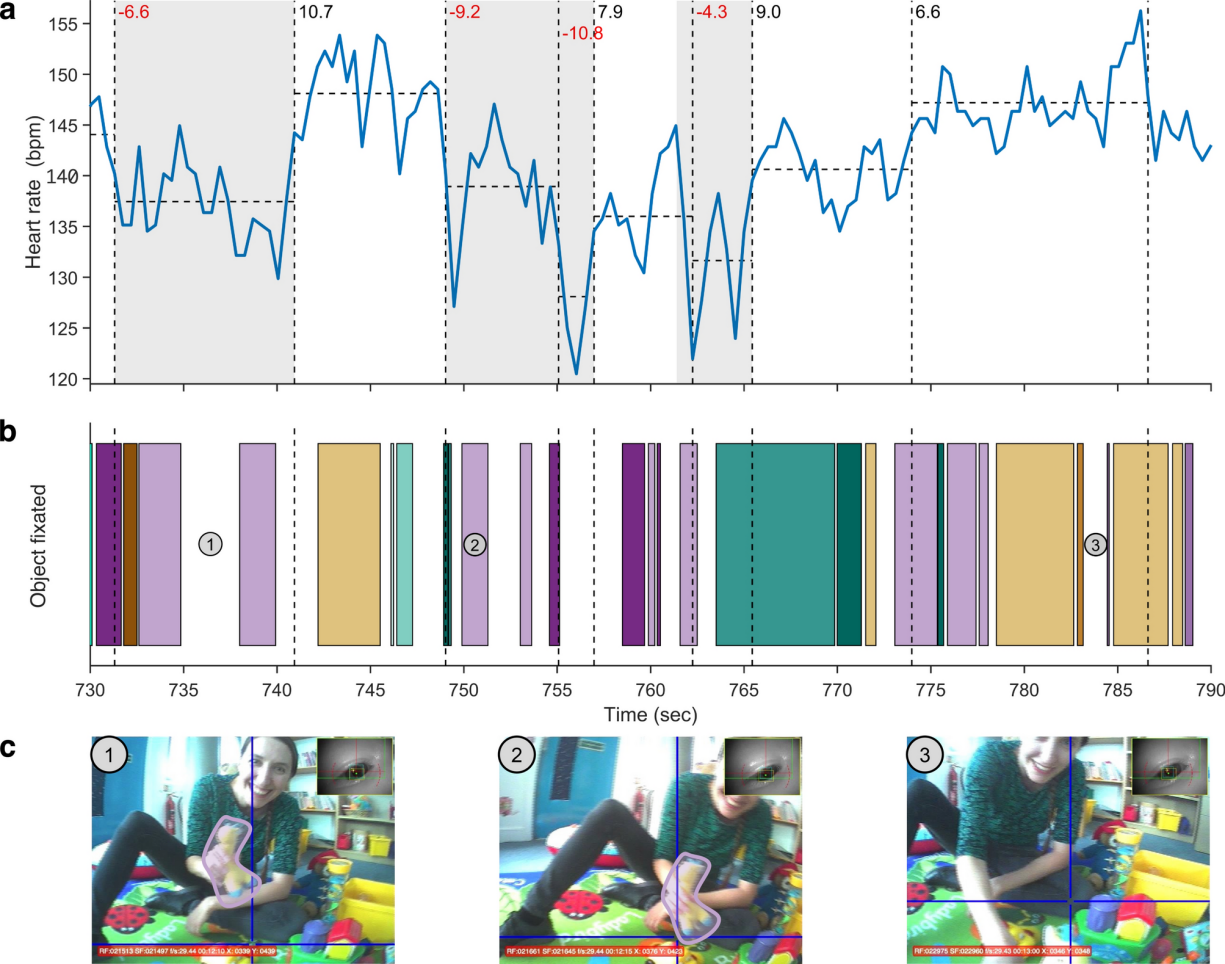
Example of human-coded sustained attention for a 6-month-old participant for a 1-min time window. (**a**) The solid blue line indicates the HR time series (in bpm). The vertical dashed black lines indicate change points which divide the HR time series into segments. The horizontal dashed black lines show the mean HR for each segment. The change in mean HR relative to the preceding segment is displayed in the top left of each segment (red indicates deceleration; black indicates acceleration). The grey regions indicate sustained attention periods. In the third grey region, the onset time was shifted to the first HR peak before the change point as the change in HR was − 4.3 bpm, with the change between the HR peak immediately before and after the change point > 5 bpm. (**b**) The fixated objects from the eye-tracking data. Each unique colour bar represents a unique object (57 total objects), e.g., purple bars represent periods of fixation on a plush giraffe. (**c**) Three representative frames from the infant’s egocentric view were extracted at each point in time corresponding to the numbered grey circles from Panel B. The fixation (blue crosshair) and participant-eye view from the eye tracker are superimposed onto each frame. The plush giraffe outlined and shaded in purple corresponds to the purple bars in Panel B (periods when the infant fixates on the giraffe).

Three experimenters (two authors) independently coded SA periods. Figure 3 illustrates the custom MATLAB graphical user interface for the protocol. First, detected change points in the HR time series were marked to create segments (Fig. 3a, vertical dashed lines). The change in mean HR of a segment relative to the preceding segment was then calculated.

The change in mean HR on consecutive segments was next screened for putative SA periods as follows. The SA onset was set to the change point when there was a decrease of at least 5 bpm in the mean HR relative to the preceding segment. If the change was between 3 and 5 bpm, the coder assessed the peak-to-peak drop around the change point. If the drop exceeded 5 bpm, the onset was set to the time of the peak immediately before the change point (e.g., Fig. 3a, third grey region). For consecutive drops meeting these criteria, the earliest drop determined the SA onset (e.g., Fig. 3a, second grey region). The termination of SA was set at the change point with an HR increase of at least 5 bpm. For 3–5 bpm increases, termination was set to the nearest HR peak after the change point if the peak-to-peak rise was 5 bpm or more. SA periods shorter than 2 s were excluded^78^.

Lastly, coders assessed whether participants were looking at or following a small number of objects during the identified putative SA periods. This was done using the fixation-label time series (Fig. 3b) and inspecting the screen video overlaid with the fixation crosshair (Fig. 3c). The experimental setup contained several toys or objects of potential interest (maximum 25), similar to a typical home^79,80^, and participants were free to move about the space. In most lab-based studies investigating HRDSA, complex and dynamic stimuli, which comprise multiple social and non-social elements and changes in scenes, were presented on computer screens^17,81–84^. In these studies, periods of HR deceleration indicative of SA have been associated with both brief and extended ‘looks’ towards the screen, each ‘look’ encompassing fixations towards different objects, as well as different scenes and events. Other lab-based studies presented infants with 1 to 6 toys, often handed to them one at a time^42,78,85^. Considering the more naturalistic conditions in our study, we set the threshold to a maximum of 5 objects to be fixated or followed in order for a period of HR deceleration to be considered SA.

To assess inter-rater reliability, we randomly selected 15 participants from the five age groups, with two of the three coders independently coding each participant. Reliability was determined by counting overlapping attention and inattention periods between the two coders, allowing for any degree of overlap. The coders showed substantial agreement (Cohen’s κ: *M* = 0.764; *SD* = 0.116; range: 0.496 to 0.895).

#### Saliency and clutter extraction

We calculated saliency and clutter from the acquired scene video frames and fixation data during the naturalistic free-play periods. These measures can attract infant and adult participants’ attention^54,61,64,86–88^. The fixation distribution per age group is presented in the Supplementary Information (Fig. S2, Table S1).

**Fig. 4:**
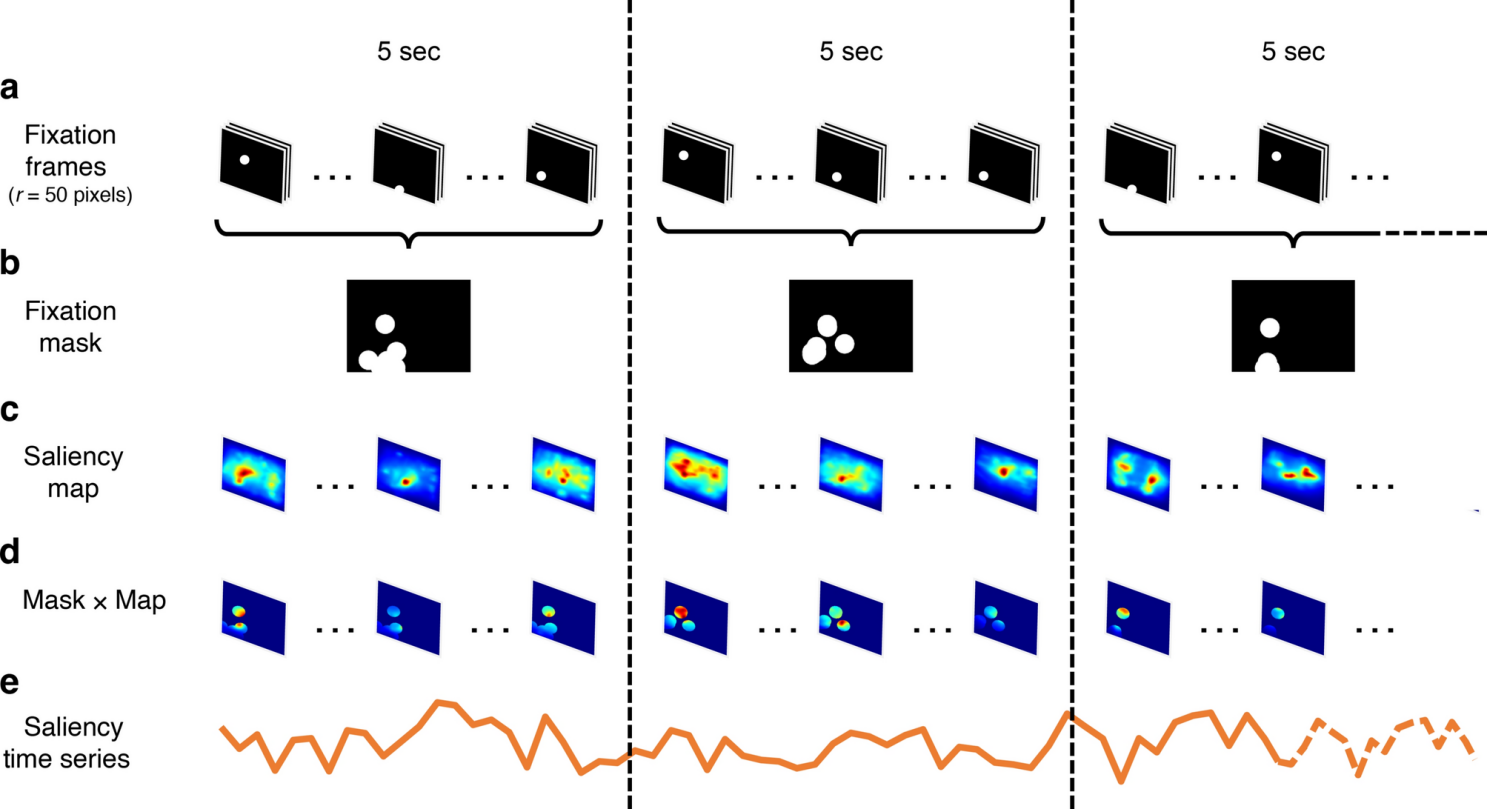
Example of a saliency time series during free play. (**a**) The fixation time series is divided into consecutive 5-s time windows (150 frames at 30 Hz). A spatial window was created around each fixation (radius, *r* = 50 pixels; white circles). (**b**) All fixations within each 5-s time window are accumulated to form a binary fixation mask (black pixels = 0, white pixels = 1). (**c**) The saliency maps within each window are extracted (15 frames at 3 Hz). (**d**) Each saliency map within a 5-s time window is multiplied by the corresponding binary fixation mask for that window. (**e**) The saliency time series is created by averaging across all pixels within the respective binary fixation mask at each time point. The same procedure is used for the clutter time series. For a detailed illustration of the binary fixation mask applied to a saliency map, see Figure S3.

Figure 4 illustrates how we generated a saliency time series (see also Fig. S3). Based on the duration of SA periods in our data, we first segmented the frames into consecutive 5-s time windows to allow for the temporal integration of fixated visual information. Second, we created a 50-pixel radius circular mask^5,89,90^ centred on each fixation and accumulated these masks within each window to create a binary fixation mask. On average, approximately six fixations contributed to each binary fixation mask, covering approximately 7% of the frame area (Table S2). Third, we extracted saliency maps from the frames, applied the binary fixation mask to each map within that window, and averaged the saliency values of pixels inside the mask to create the time series. During time windows with no fixations, the saliency/clutter values were removed from further analyses. We used the Graph-Based Visual Saliency (GBVS) algorithm to compute saliency^65^ and the Feature Congestion measure to compute clutter^66^. Full details of this procedure are provided in the Supplementary Information (5. Saliency and clutter extraction).

### The automatic sustained attention prediction (ASAP) model

**Fig. 5:**
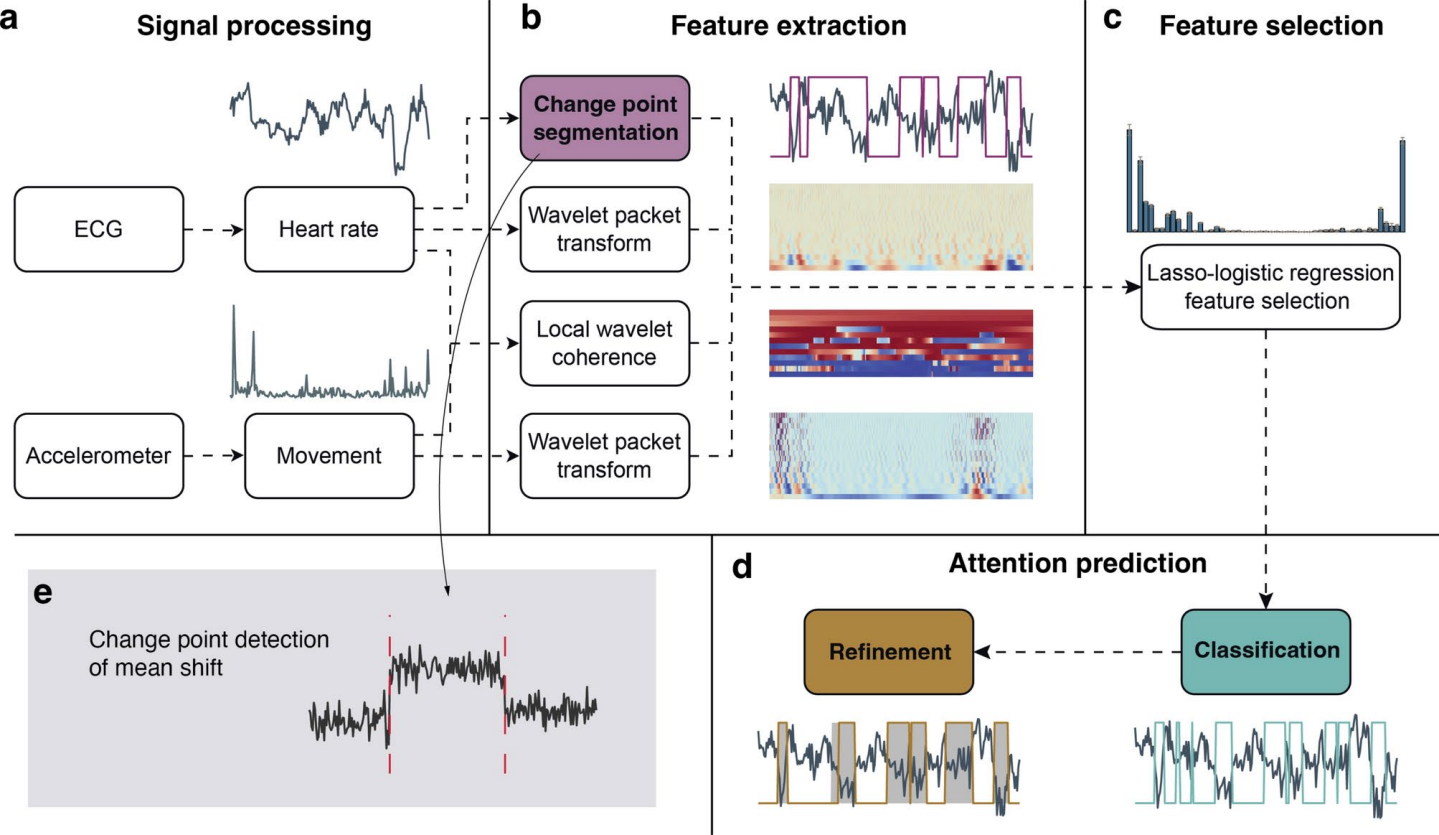
ASAP procedure overview. (**a**) Signal processing to extract heart rate (HR) and movement (acceleration magnitude). (**b**) Feature extraction to produce change point segments, wavelet packet transforms of HR and Acc, and local wavelet coherence between HR and Acc. (**c**) Feature selection using Lasso regularised logistic regression. (**d**) Attention prediction using logistic regression with selected features and further refinement to reconstruct the temporal structure. Coloured boxes in (**b**)–(**d**) indicate the three stages of attention prediction. (**e**) An example of change point detection of mean shift.

ASAP detected HRDSA by identifying abrupt changes in HR and leveraging information from HR and Acc signals associated with SA. Using the processed HR and Acc signals, we conducted feature extraction and feature selection to establish a classifier for attention prediction. As shown in Fig. 5, the ASAP procedure consists of three steps (colour-coded in Fig. 5): *change point segmentation*, *point-wise classification*, and *segmentation refinement*.

#### Step 1: Change point segmentation

Change point detection (CPD) is a statistical method that detects changes in properties (e.g., mean, variance, slope) in a time series. Here, the change point segmentation step (Fig. 3) identified boundaries of putative SA periods indicated by steep decreases and increases in the mean of HR (Fig. 5e). Given that infant free play is characterised by frequent short periods of SA and that the heartbeat defines a fundamental minimum resolution of 2–3 samples per second for observing changes, we adopted a CPD methodology that produces change points best fitting these resolution constraints: wild binary segmentation (WBS2) with a model selection criterion of the steepest drop to low levels (SDLL)^40^ (for the selection motivation, see Supplementary Information – *6. Change Point Detection*). The method is available from CRAN via the R package ‘*breakfast*’ (version 2.2), using ‘*wbs2*’ and ‘*sdll*’ options.

Applied to our dataset, this approach had an average rate of CPD of one change point per 8 to 9 heartbeats. Change points were classified as descending and ascending by calculating the local HR slope. We then applied the same rules as for the *human coding protocol* to screen and refine the boundaries of SA. Step 1 resulted in a binary time series indicating putative attention and inattention segments.

#### Step 2: Point-wise classification

**Fig. 6:**
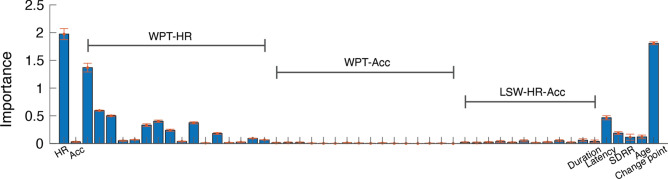
Feature importance by Lasso regularised logistic regression. Importance is measured using the absolute values of the standardised logistic regression coefficients, averaged across the fivefold cross-validation. Error bars are standard deviations across the fivefold cross-validation. Selected features had mean Importance > 0.03 (threshold determined by cross-validation). WPT-HR: wavelet packet transform of HR; WPT-Acc: wavelet packet transform of Acc; LSW-HR-Acc: local stationary wavelet estimated coherence between HR and Acc. In these cases, each bar corresponds to a frequency band.

*Feature extraction.* Time points were mainly classified on the temporal and spectral information extracted from HR and Acc and the putative SA segments identified in Step 1. Fifty-one predictor features were extracted (see Table S3 and Fig. 6 for full list). (1) The HR and Acc magnitude. (2) Time–frequency decomposition using the wavelet packet transform was used to analyse HR and Acc over different time scales (MATLAB 2022a routine ‘*modwpt’*, Daubechies wavelet with two vanishing moments), yielding sixteen features (frequency bands) for each measure (WPT-HR and WPT-Acc). (3) The time-evolving spectral cross-dependence between HR and Acc was estimated using multivariate locally stationary wavelet processes, with the localised coherence Fisher’s-z transformed (R package ‘*mvLSW*’ version 1.2.5)^91^, yielding 12 features (LSW-HR-Acc). (4) HRV measures from the recording session were included, specifically the standard deviation of inter-beat intervals (SDRR) – the time deviation between consecutive R-peaks. (5) A binary time series derived from the CPD step was included, indicating potential attention and inattention states (Change point binary). (6) Segment duration (Duration) and time to the previous segment (Latency) yielded two more features. (7) Lastly, age in months was included.

These variables formed the predictors, *X*, in the model *Y* = *f(X)*, where *Y* represents a binary time series of attention and inattention from human coders. We initially included the above features as predictors, before performing feature selection to determine the most informative features in predicting attention. All variables were sampled at 2 Hz.

*Feature selection.* The 75 participant sessions were split into training and test sets (~ 9:1), where each partition contained randomly selected sessions with approximately equal age distribution. The training set consisted of 67 participant sessions (194,360 time points), and the test set consisted of 8 participant sessions (22,367 time points). Our model selection procedure was conducted using the training set, while the test set was kept separate and only used for the final evaluation of model performance. Feature selection was performed using regularised logistic regression, which shrinks a subset of the estimated coefficients to zero by employing an L1-norm penalty (Lasso) for covariates deemed to have non-significant contributions to attention^92^ (see Supplementary Information, Sect. 7, for the mathematical formulation). The tuning parameter ($\lambda$) that controls the strength of the L1-norm penalization was determined by cross-validation ($\lambda =3.4e-5$). The regularised logistic regression model was trained using the ‘*lassoglm*’ MATLAB routine with 100% L1 penalty.

The Lasso approach preserved 24 out of the 51 feature variables (Fig. 6 and Table S3), including the HR, a subset of wavelet transforms of the HR (WPT-HR in Fig. 6; 13/16 selected; see Table S3 for selected frequency bands), duration, latency, SDRR, change point binary, and age. The Acc alone and its wavelet transform (WPT-Acc) were determined to be unimportant, but five of the twelve HR-Acc coherence bands (LSW-HR-Acc) were included. The logistic regression model was then retrained without regularisation using the training set based on the 24 features. The performance was then evaluated using the test set. Using other classifiers resulted in comparable performance (Fig. S4, Table S4).

*Model training.* Imbalanced class distribution, where one class significantly outnumbers the other, can bias model training. In our case, attention occupied about 29% of the total time. To address this, we tested whether balancing class distribution could improve model performance using fivefold cross-validation within the training set. Data balancing was achieved using the Synthetic Minority Over-sampling Technique (SMOTE)^93^. SMOTE improved the model performance based on the F1-score criterion. We showed that the model’s performance remained stable regardless of dataset size, with performance metrics plateauing when trained on more than half of the training set (see Supplementary Information, Sect. 10, for sensitivity test; Fig. S5). To optimise the model accuracy so that it can be deployed for future data collected in natural environments, we provide the final model trained on the entire dataset with SMOTE oversampling applied.

#### Step 3: Segmentation refinement

Point-wise classification (Step 2) did not preserve the temporal structure of the putative SA segments obtained in Step 1, and could disrupt long SA periods. To maintain the temporal structure, we merged adjacent segments based on the information from Step 1 and Step 2. First, segments identified in Step 2 that were 2.5 s or less apart were merged. Next, if the predicted attention in Step 2 covered more than 90% of a Step 1 SA segment, the entire segment was classified as an SA period, provided that no attention span exceeded 50 s. These criteria were established using fivefold cross-validation within the training set, which optimised on producing an attention duration distribution similar to the human-annotated distribution. The Kolmogorov–Smirnov (KS) test was applied to compare the model distribution to the human distribution.

### Model evaluation

The performance of the ASAP model was evaluated on the test set (8 sessions from all age groups of 6-, 9-, 12-, 24-, and 36- months). First, we assessed the point-wise performance of the method in comparison to human coding for different metrics: accuracy $\frac{\# of Correct Predictions}{Total \# of Predictions}$ , precision $\frac{True Positives}{True Positives+False Positives},$ recall $\frac{True Positives}{True Positives+False Negatives}$, F1-score $\frac{2\times Precision\times Recall}{Precision+Recall}$, and inter-rater reliability (Cohen’s κ) at each step. Second, the KS test was used to assess the similarity of the predicted duration distribution compared to the human-coded data. Finally, we examined whether our prediction method could preserve the natural statistics associated with attention as identified by human coding (see Model application: Cross-validation of the ASAP model with egocentric visual information section). The criterion of statistical significance was set to 0.05.

## Results

### Model performance

**Fig. 7:**
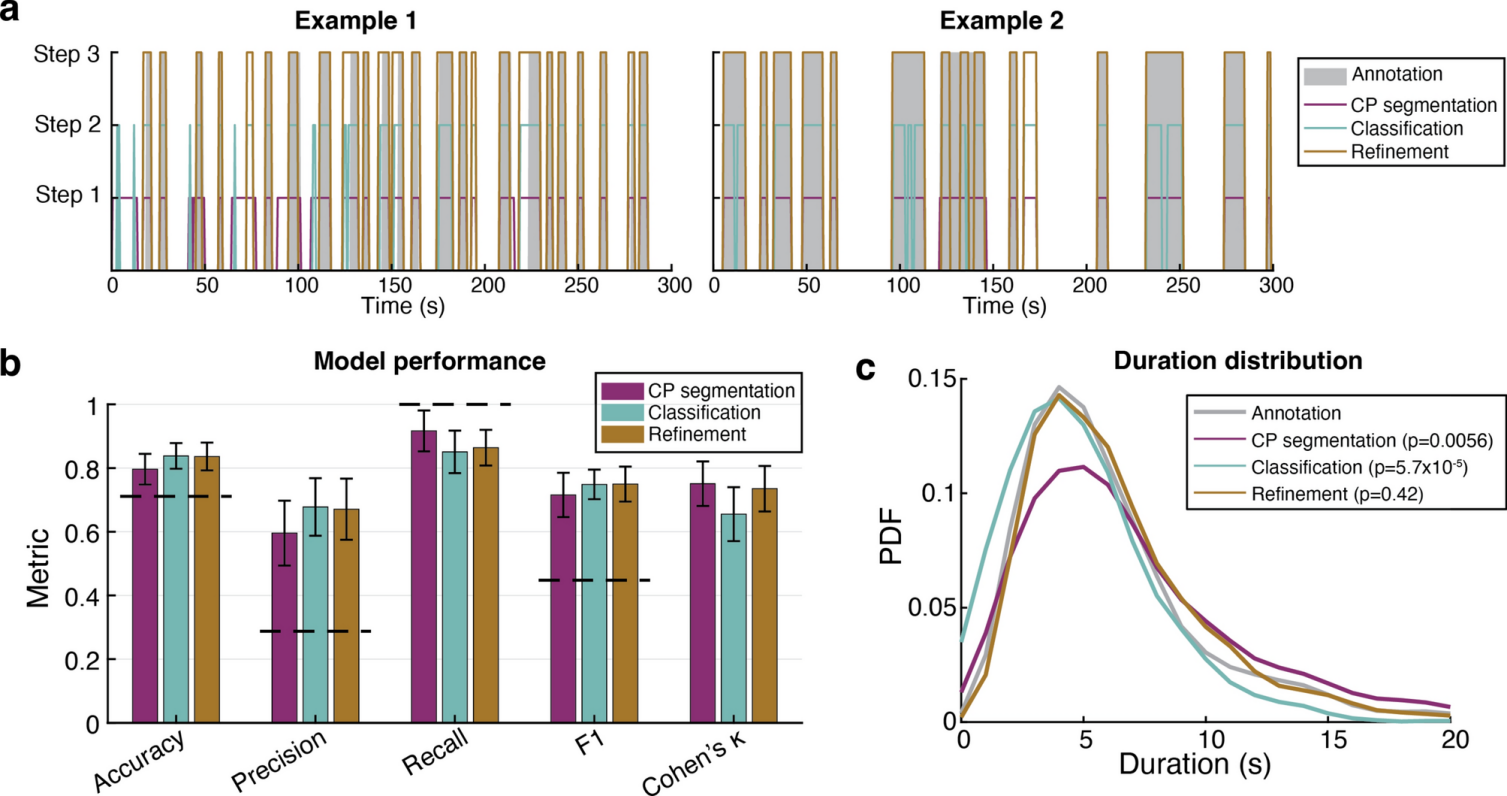
ASAP performance. (**a**) Two examples of attention prediction through 3 steps. (**b**) A summary of performance metrics. Colour codes are identical to (**a**) and Fig. 5. Dashed lines indicate null estimates. The null is obtained for accuracy when guessing all negative; for precision, recall, and F1, the null is calculated with all-positive guesses. Error bars are standard deviations. (**c**) Distributions of segment duration are obtained from each step. *P*-values revealing the distribution similarity are based on KS test. CP: change point. PDF: probability density function.

The ASAP procedure progressively approximated human-coded attention periods through the three steps (Fig. 7a, b). The change point segmentation phase (Step 1) attained a point-wise accuracy of 80 ± 5% (mean ± SD), precision of 60 ± 10%, recall of 92 ± 6%, F1-score of 0.72 ± 0.07 and Cohen’s κ of 0.76 ± 0.07. An all-negative prediction, based on the assumption that participants were not paying attention for the majority of the time, resulted in a significantly lower accuracy of 71%. Conversely, an all-positive prediction led to a precision of 29%, consequently producing a null F1-score of 0.45. The failure to achieve 100% recall can be attributed to adjustments made during the human coding process incorporating the video and fixation data. Additionally, the duration of attention segments exhibited a broader distribution than the human-coded distribution (*p* = 0.0056, KS test, Fig. 7c).

The classification step (Step 2) improved the prediction accuracy to 84 ± 4% (*p* = 0.0060, paired *t*-test between Step 1 and 2) by increasing precision to 68 ± 9% (*p* = 1.9e−4, paired *t*-test). This step reduced recall to 85 ± 7% (*p* = 0.0025, paired *t*-test), indicating a trade-off between precision and recall. Cohen’s κ also dropped to 0.66 ± 0.08 (*p* = 5.0e−4, paired *t-test*) due to the reduction in recall. Step 2 fragmented long attention periods, resulting in a duration distribution skewed towards shorter durations (*p* = 5.7e−5, KS test, Fig. 7c). Overall, Step 2 improved the accuracy of point-wise predictions but did not maintain the temporal structure.

The refinement phase (Step 3) merged short segments to reconstruct long attention spans. The point-wise performance remained largely unchanged, with accuracy of 84 ± 4%, precision of 67 ± 10%, recall of 86 ± 6%, F1-score of 0.75 ± 0.06, and Cohen’s κ of 0.74 ± 0.07. Importantly, this phase restored the duration distribution to be statistically comparable to the human-coded distribution (*p* = 0.42, KS test, Fig. 7c). Therefore, Step 3 was crucial for recovering intact attention periods. Additionally, we did not observe a significant effect of age on any of the metrics at any step (*p*s > 0.2, linear regression).

### Model application: Cross-validation of the ASAP model with egocentric visual information

An infant’s attentional state is linked to their sensory experiences within natural environments. We estimated the changes to visual saliency and clutter of fixated regions over time (see Methods—*Saliency and clutter extraction* section and Fig. 4) and compared these measurements between ASAP and human-coded attention and inattention periods, while also examining potential developmental changes. Only data from free play sessions with at least one minute of cumulative recorded fixation (*N* = 72; 966 ± 368 s) were included in this analysis. The number of fixated regions during attention (0.048 ± 0.014 fixations per frame (mean ± SD)) and inattention (0.047 ± 0.014) did not differ significantly (*p* = 0.35, paired *t*-test). We standardised saliency and clutter measures within each session to eliminate systematic variation across participants. The session means of each measure were analysed using a linear model (*fitglm* in MATLAB, 2022a), with three factors: attentional state (attention vs. inattention), data source (human coding vs. ASAP prediction), and age, along with their four two-way interactions and one three-way interaction term (see Supplementary Information, Table S5). A Bonferroni correction was applied to adjust for multiple comparisons across factors, interactions, and responses.

**Fig. 8:**
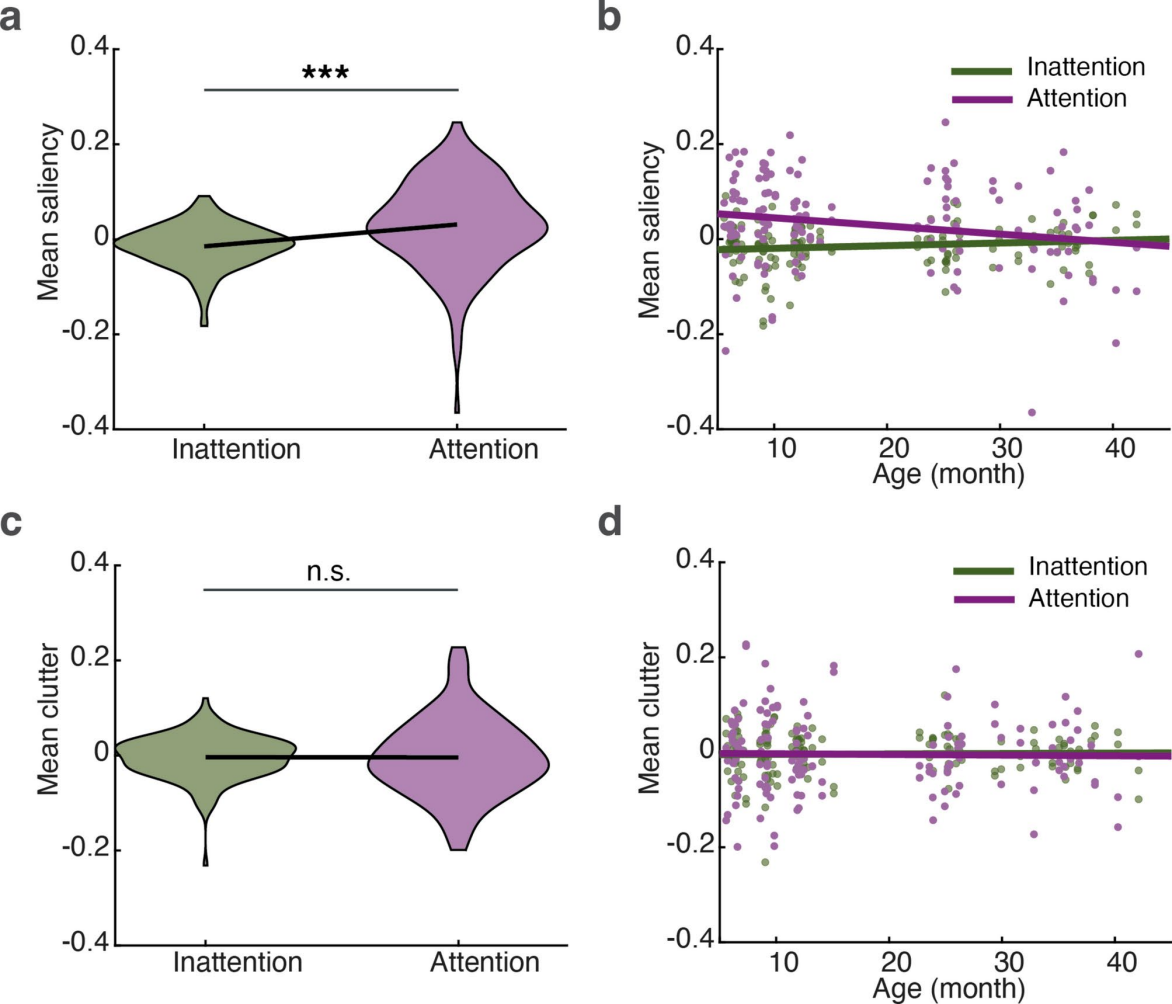
Effects of attention on visual features. (**a**) Violin plot of the attentional effect on visual saliency. (**b**) Interaction effect on visual saliency between attentional states and age, with lines fitted by linear regression. (**c**) and (**d**) respectively show the equivalent graphs for clutter.

We found that attentional state had a significant effect on mean saliency, with higher saliency of fixated regions during attention than inattention periods (Bonferroni-corrected *p* = 9.2e-7; Fig. 8a). There was also a significant interaction between attentional state and age for saliency (Bonferroni-corrected *p* = 0.0011; Fig. 8b): the saliency of fixated regions decreased with age during attention periods but remained relatively constant during inattention periods. No significant effects or interactions involving the data source were found (complete statistical results in Table S5). Additionally, no attentional or age-related effects on the visual clutter of fixated regions were observed (all *p*s > 0.05; Fig. 8c, d; Table S5).

## Discussion

There has been increasing interest in employing naturalistic approaches to study human development^18,94–97^. Sustained attention (SA), a cognitive ability that emerges during infancy, is particularly important due to its widespread implications across various developmental domains^1,3,5^. Despite its significance, most current SA developmental research relies on lab-based paradigms, with limited understanding of whether these findings extrapolate to the natural environment, or how the mutual interactions between the developing infant and their everyday environment contribute to SA emergence. In this study, we propose an innovative algorithm—ASAP—that harnesses signals recorded unobtrusively from wearable technology (e.g., the EgoActive platform^26^) to detect infant SA manifested spontaneously.

ASAP is the first model to utilize ECG and Acc signals to classify attentional states with high temporal resolution in preverbal infants and toddlers, making it particularly well-suited for settings outside the laboratory, where the environment changes continuously and infants are free to move around. Previous efforts for using machine learning methods based on physiological signals (e.g., ECG, EEG) have exclusively focused on predicting attentional states in adults^98–102^. These studies have predominantly used well-controlled lab-based paradigms, with the ground truth established based on adult participants’ self-reported attentional states or the nature of the stimuli/task, usually over longer timescales (several minutes). Other applications of ECG signals in machine learning have aimed to diagnose neurocognitive disorders such as ADHD^103^ or autism^104^. Similar to our approach, these studies employed feature extraction, including spectro-temporal decomposition of physiological signals, to train classification models. However, unlike our study, these models were not trained to predict attentional states or specific cognitive states, but rather to predict specific diagnoses. Furthermore, an important innovation of our approach is the integration of change point detection, which enables the robust and efficient classification of densely sampled attention and inattention periods. This innovation provides a powerful tool to capture and understand infants’ attentional state in response to a dynamically changing environment.

The key HR features contributing to ASAP’s successful SA detection are the HR deceleration during periods of SA relative to those of non-attention^35^, and the HR fluctuations in both time and frequency domains^45–48,50–53,105^. The feature importance analysis revealed that HR fluctuations in the 0.03–0.15 Hz and 0.3–0.6 Hz frequency bands contributed significantly to predicting SA (Fig. 6 and Table S3), corresponding to the two distinct peaks previously observed in the infant HR power spectrum^106^. A reduction in body movement has also been previously used as a criterion for determining attention^107,108^, and associations have been shown between changes in HR and body movement linked to measures of attention^70,71,109,110^. While previous studies have considered the movement of the head or limbs^70,71^, the feature selection in this study did not identify the torso Acc magnitude as playing a significant role in predicting SA. Our choice for relying on the torso movement was motivated by both empirical and pragmatic reasons. Empirically, comprehensive studies with non-human primates show that when considered alongside movement of the head and limbs, torso movement is most strongly coupled with the HR^53^. Pragmatically, in many of the wearable sensors typically used for naturalistic recordings, accelerometers are placed on the torso^26^. Although Acc magnitude did not predict SA in this study, its coupling with cardiac activity did, especially at very low frequencies below 0.1 Hz (Table S3). This frequency range aligns with previously reported spontaneous brain fluctuations that are associated with arousal and its coordination with movement in humans and non-human primates^53,111,112^. Considering that some of the key features in defining periods of SA are represented by the deceleration and acceleration of the HR, as well as HR fluctuations in the frequency domain, our findings suggest that these features are less likely to be the direct result of the changes in torso movement.

We also investigated whether the variability in performance across sessions was driven by intrinsic dataset characteristics or biases inherent to the model. By analysing the correlation between human coder agreement and the agreement between human coders and the ASAP model, we found that intrinsic factors influenced performance for both (Supplementary Information, Fig. S6a, b). Further analysis indicated that SDRR (an HRV measure) was a key intrinsic feature that contributed to SA classification performance (Fig. S6c). Previous studies have shown that respiratory sinus arrhythmia (RSA), a separate HRV measure within the respiratory frequency range, predicts the extent of HR deceleration entering the attentional phase^76^. Individuals with higher RSA levels (also higher SDRR) are more resistant to distraction^46,76^. Therefore, higher HR variation may lead to more distinct HRDSA, potentially reducing the confusion in the attention classification task. This correlation supported the inclusion of SDRR as a feature in the attention prediction model, and our feature selection analysis further validated its relevance. Overall, these supplementary analyses demonstrate that the ASAP model did not have any inherent bias leading to systematic errors, and that errors in model classification align with discrepancies between human coders.

We applied ASAP-labelled data to study visual attention development by leveraging the egocentric video and fixation data and generating the perceptual features of visual saliency and clutter. Three key findings emerged. First, and of critical importance, the results were similar for the periods of attention determined by human coders and those detected by ASAP (Table S5). This demonstrates that implementing ASAP can be a less costly (time and human resources) approach, making it ideal for studies involving large data recorded in the natural environment or in the lab. Second, similar to some previous studies^113^, during SA periods, infants fixated on areas with higher perceptual salience than during periods of inattention. Older infants were observed to fixate on less perceptually salient regions during sustained attention than younger infants. This could reflect the older infants’ increased reliance on more high-level properties of the scenes, although further consideration of other factors (e.g., local meaning) that tend to covary with salience would be required to more definitively conclude this effect^61^. Third, unlike some of the previous studies^114^, the areas fixated during attention and inattention did not seem to differ in terms of visual clutter, irrespective of infants’ age. In part, this could be due to the fact that here we considered the amount of fixated feature congestion, which may be less relevant for allocating and sustaining attention compared to how cluttered the entire scene is^114^. Our results help to replicate and extend findings from lab-based studies under constrained conditions to more naturalistic scenarios. Importantly, for the first time, the relation with visual saliency is reported for HRDSA, supporting the interpretation that visual saliency not only influences what infants are likely to orient towards but also what is likely to be processed in depth^73,115^.

It is interesting to note that infants’ and toddlers’ capacity to control the environment can reduce visual clutter in their egocentric view. Older toddlers are more likely to be able to control their visual clutter, whether by interacting with objects directly or by moving their body, head, or eyes. These physical interactions with their environment can also change the visual saliency from their egocentric view. By comparison, caregivers are more likely to move objects in younger infants’ environments as infants may have less motor control than older toddlers. We did not find a main effect of age on the overall levels of visual saliency or feature congestion (*p*s > 0.14, see Supplementary Table S5). These null findings may relate to individual differences in motor development, for instance; and these differences may possibly be larger than age differences^116–118^. Thus, considering both age and individual differences can be an exciting avenue of future research.

### Limitations and future research

ASAP is based on HR-defined visual attention. Our model yields a weaker precision (~ 67%) relative to the other metrics, which may be partly due to other events inducing similar HR changes. Attention in other sensory modalities (e.g., audition) can also be accompanied by HR deceleration^119^, resulting in an HR waveform similar to that observed during visual attention. Additionally, vocalisations have been associated with an HR dynamic that mirrors the one observed during visual SA^53,105^. Other information can help differentiate these possibilities from visual SA (the ground truth in our study), and future work could extend ASAP to detect sustained auditory attention while also incorporating infants’ and toddlers’ vocalisation as features^120^, potentially improving its performance.

This study represents the first step in developing the ASAP algorithm based on a methodological lab set-up that includes both structured and unstructured activities, some of which emulate those carried out at home (e.g., free play on a mat with toys). This step is crucial to ensure the reliability of the algorithm. Future research may consider data recorded in home settings, with a methodological set-up adequate for this context (e.g., wireless wearable sensors such as the EgoActive^26^, a combination of home- and lab-based assessments, as well as assessments of attention based on parental reports^121^).

It would also be interesting to explore the possibility of developing new algorithms that complement ASAP to predict other attentional processes. Studies that integrate eye-tracking and ECG to investigate infant attention responses to emotional stimuli have shown strong associations between a lower frequency of saccades away from emotional faces and concomitant HR deceleration as an index of attention-orienting responses^122^. These findings raise the possibility that ECG signals may also have the potential to be used as predictors for attention orientation and eye-movements.

## Conclusions

We demonstrated that ASAP achieved solid performance in the challenging task of detecting periods of sustained attention based on HR and Acc signals across the age range and conditions considered here, including natural, free-play behaviours. This is critical as HR and Acc are the predominant signals recorded with unobtrusive wearable sensors for infants and are invariant across platforms. Although future work can expand ASAP to include other sensory modalities and increase its performance metrics, it can currently provide a powerful tool to detect visual sustained attention in natural settings (e.g., home), where infants and young children are free to move. Together with available wearable technology, ASAP provides unprecedented opportunities for understanding how attention develops in the context of infants’ and toddlers’ daily experiences, and to support explanations of attention development with high ecological validity.

## Supplementary Information

Supplementary Information.

## Data Availability

All new data and code required to reproduce the main results and figures of the article are available at: https://github.com/yisiszhang/ASAP. The raw video files cannot be shared in order to protect participants’ privacy and confidentiality, in line with data protection legislation.
