# Supplementary material for: An automatic sustained attention prediction (ASAP) method for infants and toddlers using wearable device signals
Source: Sci Rep. 2025 Apr 17;15:13298. doi: 10.1038/s41598-025-96794-x (PMC12006380; doi:10.1038/s41598-025-96794-x)
Supplement: Supplementary file 1 — Supplementary Information. [file 41598_2025_96794_MOESM1_ESM.pdf]

Supplementary Information

1. Tasks description

The experimental session was divided into two parts. Part 1 consisted of the initial setup, synchronisation of the devices, and the eye-tracking calibration procedures, and took place in the laboratory room. The experimenters first fitted the eye tracker and body sensor on the participant. The recordings from the eye tracker and body sensor were then temporally synchronised by turning on and off the room light. Once the participants were at ease, *peek-a-boo* and follow-the-toy games were used to collect calibration points for the offline calibration of the eye-tracker. Five calibration points were collected as part of the *peek-a-boo* game. Using a blackboard with five cutout windows (located in the four corners and centre), the experimenter showed and hid a red dinosaur hand puppet and tried to draw participant's gaze towards the puppet. The puppet was randomly shown several times at the five spatial locations[1]. The participants sat approximately 3.5 m from the blackboard throughout this step. During the *follow-the-toy* game, the experimenter sat close to the participant and directed their gaze to a series of colourful squeaky toys located at hand level[2] and other points covering space along the horizontal (azimuth), vertical (elevation) and depth axes not otherwise captured during the *peek-a-boo* game.

Part 2 consisted of three tasks aimed at eliciting visual attention in a variety of situations, as shown in Fig. S1. It included more structured situations (the *check-this-out* game and the *spin-the-pots* task) and more naturalistic scenarios (free play). All participants were involved in the *check-this-out* game and free play. Since the *spin-the-pots* task was designed for older infants, only 24- and 36-month-old infants participated. The *check-this-out* game consisted of a period of semi-structured play. Parents and participants were invited into a play area and asked to find a comfortable position near the centre of a play mat, similar to what they would typically do at home for play. One of the experimenters then pointed to and showed a series of six target toys strategically arranged amongst other toys on a shelf located about 3.5 m in front of the child (Fig. S1). The experimenter showed the target toys twice in the same sequential order to all participants

while trying to capture their interest. The toys and their locations were consistent across all participants. This task lasted 91 s on average (*SD* = 25 s).

The *spin-the-pots* task tests working memory[3,4]. In this task, children must find six stickers hidden under eight pots of different colours (Fig. S1b). The location of the pots is hidden from view and rotated 180 degrees after each trial. During each trial, the child can only open one pot from the eight available locations. An error occurs when the child reaches for an empty pot. This task lasted 415 s on average (*SD* = 130 s).

For free play, parents and participants could play with the toys available in the play area, move around, and/or interact with each other as they would typically do at home (Fig. S1c). This unstructured playtime lasted an average of 1120 s (SD = 359 s). During unstructured play the collection of toys was the same for all participants. However, each child-parent dyad was found to differ in their selection based on their interests.

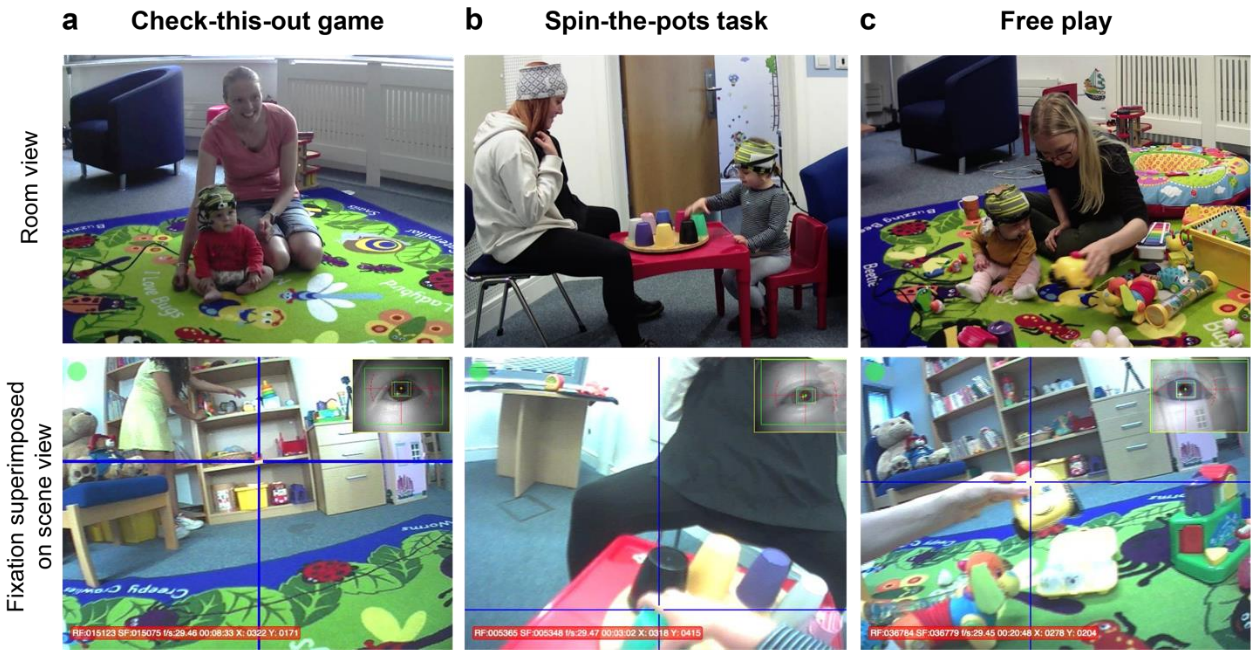

Figure S1. Examples of video frames from (a) the *check-this-out* game, (b) the *spin-the-pots* task, and (c) free play. The top row displays the room view, the bottom row shows the eye-tracking data from egocentric views.

2. Head-mounted eye-tracker calibration protocol

We followed an offline calibration protocol recommended for naturalistic tasks[5,6], using the 15 calibration points acquired at the beginning of the experimental procedure (see Supplemental Material – section 1). After the experimental session, these calibration points were used to fit the corresponding coordinates in every frame taken by the scene camera. We implemented a quality threshold of >0.80 to ensure a good fit estimation in the eye-to-scene calibration, as recommended by Yarbus software.

3. ECG signal preprocessing and HR extraction

Initial ECG processing followed the pipeline laid out in[7]. This is based on using the Neurokit2 Python package[8] default method with an additional 15 Hz high-pass frequency filter in preprocessing and local peak correction after. No square wave noise was exhibited by the Biosignalsplux sensor, so no square wave filter was used. After processing and peak detection, the initial list of peaks was parsed. Peaks were then shifted/added/subtracted by hand in order to form an accurate final list of peaks.

HR processing was done according to the protocol for clean HR datasets described in[7]. Specifically, a local median filter of width 11 with an activation threshold of 1.7x the median was used to detect outliers in the data (including the gaps in time series caused by Bluetooth dropout). The HR value within any detected gap (median duration: 1.2s) was filled in by linearly interpolating between the intervals of the nearest time point on either side of the gap that fell within the threshold. Signal quality index analysis[7] was found to have minimal effect on the processing due to the high quality of the peak detection, and so was not used.

4. Fixation Distribution During Play Period

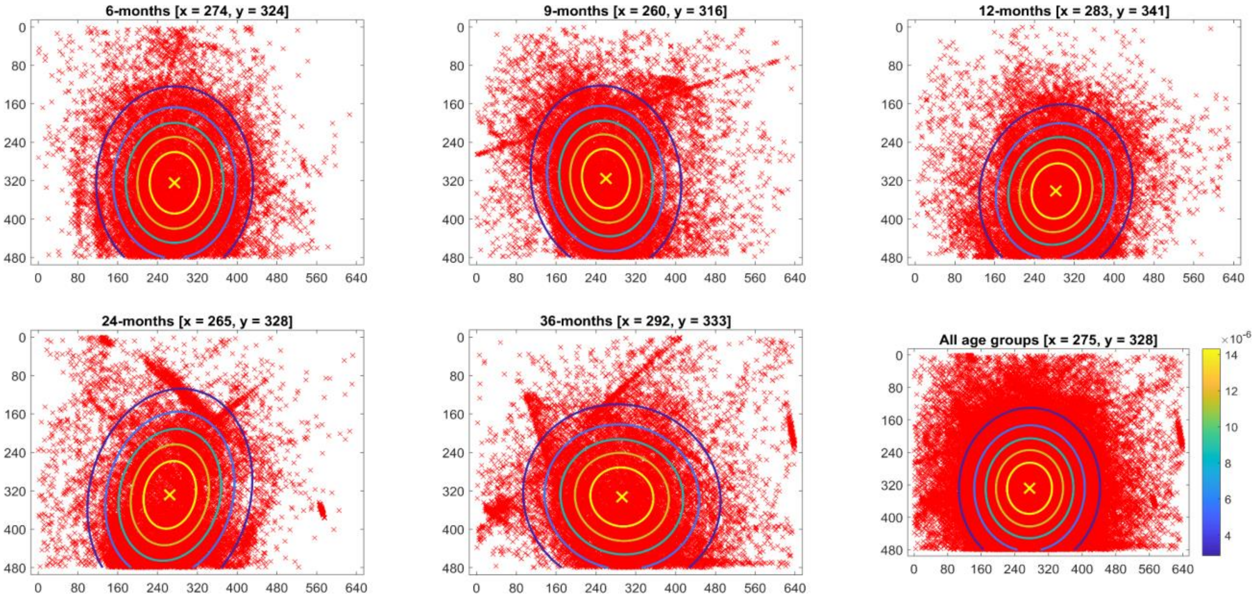

Figure S2. Fixation distribution as a function of age group. Red x's represent individual fixations, and the yellow x represents the mean fixation (indicated by the *x*- and *y*-coordinate in plot title). A multivariate normal probability density function was fitted to each distribution, with the means and covariance matrix of the *x*- and *y*-coordinates. The contours represent equal-height isolines (5 levels) of the fitted probability density function (colour bar), with fixations more likely towards the mean fixation coordinates (yellow x and contour). See Table S1 below for additional statistics.

Age Group	Total # Fixations	Fixation Distribution			Mean Play Duration (SD)
		Mean X (SD)	Mean Y (SD)	cov(X,Y)	
6-months (N = 15)	17881	274.3 (83.3)	324.4 (106.1)	-59.9	971.1 (335.7)
9-months (N = 15)	23087	260.1 (82.7)	315.5 (102.1)	581.0	1223.9 (365.5)
12-months (N = 15)	21486	283.0 (81.4)	341.4 (95.1)	-614.7	1168.9 (254.6)
24-months (N = 16)	18600	264.8 (87.5)	328.4 (116.7)	-1439.6	1042.1 (415.2)
36-months (N = 14)	21223	292.1 (104.8)	332.7 (101.8)	470.1	1203.9 (377.4)
All age groups (N = 75)	102277	274.9 (88.7)	328.4 (104.5)	-92.0	1119.8 (358.7)

Table S1. Fixation distribution as a function of age. The x- and y-coordinates are in pixels (frame size: 640 x 480 pixels). Play duration is in seconds. The standard deviation (SD) is in brackets. The $cov(X, Y)$ is the covariance between the x- and y-coordinates across all fixations.

5. Saliency and clutter extraction

We created 1-dimensional saliency and clutter time series based on fixated regions for each participant and investigated the extent to which attention periods predicted these time series. Here, we detail the procedure for visual saliency. The same procedure was used to generate the clutter time series. The fixation data consisted of a time series (frames) of x- and y-coordinates as computed by the Positive Science software. First, for each fixation, we created a circular mask centred on the fixation with a radius, r = 50 pixels. This radius reflects the precision of the eye tracker and allows for spatial summation of visual information around the fixation. The radius is based on previous studies that used screen-based studies with the same system[9-11]. We then segmented the fixation frames into consecutive 5-sec windows. We used a temporal window to reflect how infants and toddlers may integrate visual information at fixation across a short temporal period. The value was partly based on the duration of attention periods we observed in the data. Second, all circular masks within each time window were combined to create a binary fixation mask (see Table S2 for the

characteristics of binary fixation masks as a function of age). Third, we extracted saliency maps from the video frames. For each 5-second time window, we multiplied each saliency map in the window by the same fixation mask from that window (see Fig. S3 for an example of a binary fixation mask applied to a video frame and saliency map). Finally, we averaged the saliency across all pixels within the fixation mask to create a 1-dimensional saliency time series. There were 5-second time windows with no fixations; in these cases, the saliency/clutter values were removed from further analyses.

We used the Graph-Based Visual Saliency (GBVS) algorithm to compute saliency maps[12], as evidence suggests that this algorithm predicts infant and adult fixations better than other saliency measures[13,14]. GBVS is implemented in MATLAB by Harel and colleagues (archived at: https://github.com/Pinoshino/gbvs). We used the saliency map based on intensity (grayscale level), colour, orientation, and motion feature maps. The individual feature maps were combined using equal weights and then normalised to a value between 0 and 1. For the parameter settings, we used a feature map resolution level = 3; the Derrington-Krauskopf-Lennie colour space; four Gabor-filter orientations (0°, 45°, 90° and 135°); and four motion directions (0°, 45°, 90° and 135°). Default settings were used for all other parameters. Previous developmental studies included flicker and motion as dynamic cues[15-17]. However, for the purpose of demonstrating the utility of the ASAP model for natural environments, we focused on motion cues. Thus, we did not include flicker as (1) we did not want to reduce the weight of the motion conspicuity map by the addition of a flicker conspicuity map; and (2) we did not want to include flicker-induced apparent motion[18].

For visual clutter, we used the image-based Feature Congestion measure proposed by[19] and implemented in MATLAB (https://dspace.mit.edu/handle/1721.1/37593). This measure computes clutter based on the local variance of several features at multiple scales. These features include colour (CIELab colour space), intensity, and orientation contrasts. Default parameter settings were used.

We down-sampled the video data to 3 fps from 30 fps (i.e., every ten frames). We reduced each frame dimension by 50% (i.e., 320 x 240 pixels) using bicubic interpolation to manage computer memory and computation time. The saliency and clutter map were computed for each frame in the down-sampled and

resized video data. The motion feature map for saliency was based on the pixel grayscale difference between every ten frames (~333 ms).

	**Binary Fixation Mask Characteristics**	
**Age Group**	**Mean # Fixations (SD)**	**Mean % Frame Area (SD)**
6-months	6.6 (2.3)	8.1 (1.8)
9-months	6.7 (2.6)	8.1 (2.5)
12-months	6.5 (2.2)	7.6 (1.6)
24-months	6.1 (2.4)	7.3 (1.9)
36-months	6.7 (1.8)	8.4 (2.1)

Table S2. Characteristics of binary fixation masks as a function of age. The mean number of fixations used to create the binary fixation mask for each 5-sec time window. The mean percentage of the video frame area taken up by the mask (640 x 480 pixels). The standard deviation (SD) is in brackets.

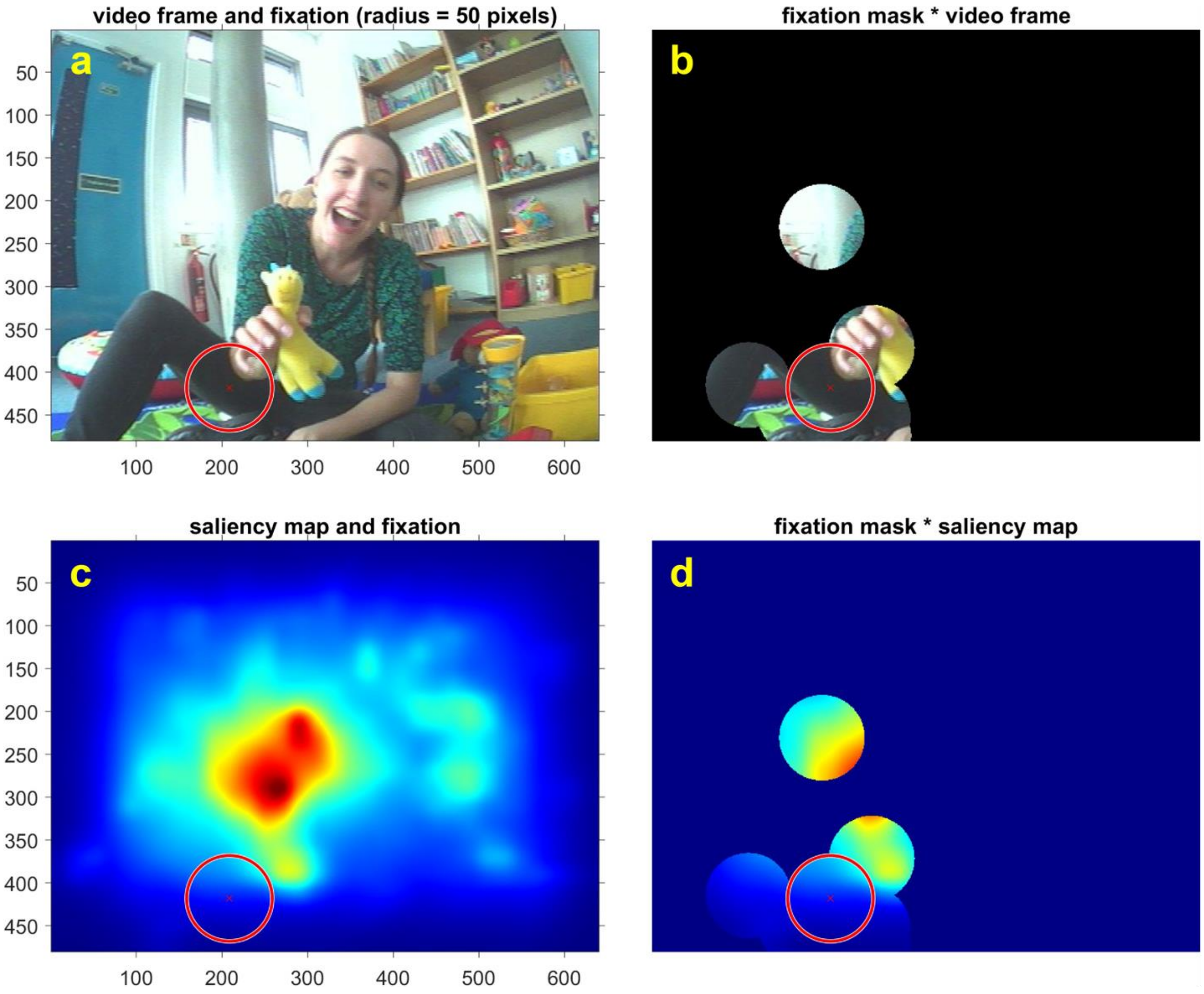

Figure S3. Example of a binary fixation mask applied to a video frame and saliency map. (a) Video frame (time point) with a fixation. The red x is an example fixation, and the red circle represents the radius (*r* = 50 pixels) around that fixation. The same fixation is illustrated in B, C and D. (b) An illustration of applying the binary fixation mask to the video frame. The mask is formed by combining 10 fixations (some fixations overlap each other). (c) The saliency map for the video frame. (d) An illustration of applying the binary fixation mask to the saliency map. The mean saliency for this frame (time point) is the average across all the pixels within the binary fixation mask.

6. Change Point Detection

The Wild Binary Segmentation2 approach

Typical change point searches amount to the iterative application of algorithmic steps that evaluate a cost function which assumes some data feature knowledge while also embedding a penalty for model complexity (see [20], and references within). Methods pertaining to (wild) binary segmentation have also been proposed and proved to be popular in conventional setups for signals with infrequent changes[21]. These approaches are designed to detect the most prominent change point and then to reiterate this procedure on the data to its left and right. However, certain data contexts are characterised by the presence of many change points, e.g., economic indicators, such as house price indices; this is also true for our data context here, characterised by the short spans of attention that infants are typically capable of exhibiting. Methodologies driven by common or even adaptive penalties, as well as binary segmentation, have been shown to completely crumble under data scenarios that feature many change points (see [22] section 2.1). A way to bypass this issue is to propose a new approach for wild binary segmentation (WBS2) and use a different model selection criterion, namely the steepest drop to low levels methodology (SDLL). This approach is shown to yield excellent results under testing data scenarios pertaining to frequent change point setups[22].

The WBS2 has been developed stemming from the WBS[21] to specifically address the many change point issues. In order to detect a putative change point in a stretch of data $X_s, \dots, X_e$ (acronyms are for 's'=start and 'e'=end), a binary segmentation (BS) approach computes a CUSUM-type 'locator' statistic over the segment $[s, e]$ and retains the location $s \le b < e$ that maximises this statistic. Mathematically, the true, unknown signal f, where $X_t = f_t + \varepsilon_t$, is considered to potentially exhibit a change point in $f_s, \dots, f_e$ at f_b for $b = argmax_b |\ CUSUM_{s,b,e}(X)\ |$, where the CUSUM statistic over the observations in the interval $[s, e]$ is defined at each location b in that interval as

$$CUSUM_{s,b,e}(X) = \sqrt{\frac{e-b}{(e-s+1)(b-s+1)}} \sum_{t=s}^{b} X_t - \sqrt{\frac{b-s+1}{(e-s+1)(e-b)}} \sum_{t=b+1}^{e} X_t$$

The WBS relies on subsampling the data $X_1, \dots, X_T$ over time intervals $[s, e]$ that are independently, randomly drawn with replacement and giving rise to M subsamples $X_{s_m}, \dots, X_{e_m}, m = 1, \dots, M$. The WBS

introduces a further step in which the above maximisation of the CUSUM-locator statistic is computed across the M randomly pre-drawn intervals that satisfies $[s_m, e_m] \subseteq [s, e]$, hence furthermore $b = argmax_{s_m,b,e_m} \mid CUSUM_{s_m,b,e_m}(X) \mid$. The identified location is then considered to be a change point if its corresponding CUSUM value exceeds the universal threshold[23], $\sigma\sqrt{2\,log\,T}$, with the noise standard deviation (σ) estimated using the median absolute deviation (MAD) across the entire dataset $X_1, \ldots, X_T$. Note that the use of thresholding is heavily reliant on a good estimator of σ and when dealing with data that features frequent change-points, this becomes a problem for the performance of WBS. If the putative change-point is deemed significant, then the steps above are re-iterated to its left $[s, b]$ and right $[b+1, e]$ intervals, using the originally drawn subsamples.

When applied to data featuring frequent change points, WBS methodology is required to address (i) the incompleteness of the solution path induced by the use of pre-drawn intervals and (ii) a new model selection strategy. The solution, referred to as WBS2, (i) adaptively uses drawn intervals at each stage based on the location of the detected change-point candidate that maximises the absolute values of the CUSUMs, hence yielding a complete solution path (each time location is a potential candidate in the solution path), and (ii) selects the appropriate change-point using a 'Steepest-Drop to Low Levels' criterion (see [22] for a complete description).

Choice of Changepoint Detection methods

The framework for change point detection consists of modelling the observed (heart rate) data $X_1, \ldots, X_T$ as a noise-contaminated version of a deterministic piecewise constant function f defined over the interval $[1, T]$ whose number of change points (N) and their precise locations are unknown. Their reliable estimation is the scope of change point detection procedures across the statistical literature[24]. Mathematically, this amounts to formalising a model for the observed data $X_t = f_t + \varepsilon_t$, with the noise ε_t assumed to follow a $N(0, \sigma^2)$ distribution, which albeit simple still poses major statistical problems, particularly when N is large (i.e., there are frequent change points in the true signal f)[22].

For the work in this paper, 37 separate change point approaches were tested from four separate packages (one from Python, three from R). These were all individually run on a subset of the data (N = 16). The optimal approach was chosen qualitatively by considering the density and positioning of change points across the signal. The heart rate will naturally change in response to a variety of stimuli in addition to attention, and so a method that slightly overpopulates change points was preferred to a more conservative approach. Many approaches use the PELT approach (Pruned Exact Linear Time)[25], but the precise choice of algorithm and penalty varies between packages.

From the Ruptures Python package (version 1.1.7)[24], the PELT model was used in combination with three penalty types - an L1 model (capturing changes in the median heart rate), an L2 model (capturing changes in the mean heart rate), and a custom model looking at linear changes in slope. All used a $log(L)\sigma^2$ penalty value, where L is the length of the signal and σ^2 is the variance.

The Changepoint R package (version 2.2.3)[26] uses a PELT algorithm with parametric cost functions. Three approaches were considered from this package, looking at changes in mean, variance, and a combination of mean and variance.

The changepoint.np R package (version 1.0.3) is an extension of the changepoints package that uses the PELT algorithm with nonparametric cost functions based on the empirical distribution of the data but allows for multiple options for penalty. Seven approaches were used, which all considered separate penalties. One approach used no penalties; two approaches used hard penalties (10 and 100), and the other four used penalties criteria- AIC (Akaike information criterion)[27], SIC (Schwarz information criterion)[28], MBIC (modified Bayes information criterion)[29] and Hannan-Quinn[30] (Package reference - https://www.jstatsoft.org/article/view/v058i03).

The last package considered was the breakfast R package (version 2.2), which uses a two-stage procedure combining a solution path generation with model selection. Four solution paths were combined with six models to produce 24 approaches. The solution paths were: SDLL (steepest descent to lowest levels)[22], Strengthened Schwarz information criterion[31], localised pruning[32], and thresholding. The six models were:

WBS (Wild Binary Segmentation)[21], WBS2[22], iDetect[33], Sequential iDetectSeq[33], Narrowest-Over-Threshold[31], and TGUH (Tail-Greedy Unbalanced Haar)[34] (Package reference https://rdrr.io/cran/breakfast/man/breakfast-package.html).

Out of all of these options, the use of SDLL solution path with WBS2 model in the breakfast R package was chosen due to producing an appropriate density of changepoints for the problem of attention estimation, and also after inspection of these change points by psychologists to confirm that those heart rate changes detected by the approach were of a suitable magnitude.

Other than the nonparametric change point approach with no penalty, almost all other methods were more conservative in change point production than SDLL with WBS2. The choice of SDLL for the solution path was found to be more impactful than the choice of model. The sequential iDetect and TGUH approaches were similar in terms of change point production when combined with the SDLL path, with the WBS2 approach being chosen as the option the package creator recommended for best performance for SDLL. The linear Ruptures approach produced very good localization of change points, but was too sparse for the purposes of attention. Some of the nonparametric change point penalties (MBIC, AIC) were also very close in terms of performance.

7. Feature selection using Lasso regularised logistic regression

Feature selection was carried out using Lasso regularised logistic regression, with the following mathematical formulation:

We define a binary response variable Y that takes the value 1 if the time point i corresponds to attention and 0 otherwise, and model the corresponding probability of attention, denoted by p_i, as $logit(p_i) = X_i^T \beta$, where $logit(p) = \frac{p}{1-p}$, X_i is the (column) vector of covariates corresponding to time i, and β is the (column) vector of unknown feature coefficients. Using a suitable transform of the data (denoted here by Z and weighted appropriately through a weighted version of the L2 norm, see [35], Section 3.1.2 for a full description), the Lasso approach for the estimation of the regression coefficients yields penalised estimates

$$\hat{\beta} = argmin_{\beta}(\| Z - X^T\beta \|_{w;2}^2 + \lambda \| \beta \|_1)$$

that are exactly zero for those covariates deemed to have insignificant contributions to attention. The estimator sparsity is controlled by the tuning parameter λ, which can be chosen via cross validation.

8. Feature selection

Feature selection was conducted using the absolute coefficients from the Lasso regularised logistic regression model. The L1 penalty in Lasso encourages sparsity by setting some coefficients to exactly zero, enabling automatic feature selection. To ensure consistency, we normalised the candidate features and applied 5-fold cross-validation. The mean absolute coefficient values across the folds were used as the criterion for feature selection. An optimal threshold was determined, above which features were selected, with the goal of maximising the F1-score across different threshold values.

#	Feature name	Absolute coefficient*	Notes
1	**HR**	1.975	
2	Acc	0.030	
3	**WPT-HR-1**	1.369	0.0312 Hz
4	**WPT-HR-2**	0.595	0.0938 Hz
5	**WPT-HR-3**	0.500	0.1562 Hz
6	**WPT-HR-4**	0.051	0.2188 Hz
7	**WPT-HR-5**	0.068	0.2812 Hz
8	**WPT-HR-6**	0.332	0.3438 Hz
9	**WPT-HR-7**	0.402	0.4062 Hz
10	**WPT-HR-8**	0.239	0.4688 Hz
11	**WPT-HR-9**	0.038	0.5312 Hz
12	**WPT-HR-10**	0.374	0.5938 Hz
13	WPT-HR-11	0.009	0.6562 Hz
14	**WPT-HR-12**	0.181	0.7188 Hz
15	WPT-HR-13	0.016	0.7812 Hz

#	Feature name	Absolute coefficient*	Notes
16	WPT-HR-14	0.026	0.8438 Hz
17	**WPT-HR-15**	0.093	0.9062 Hz
18	**WPT-HR-16**	0.066	0.9688 Hz
19	WPT-Acc-1	0.011	0.0312 Hz
20	WPT-Acc-2	0.013	0.0938 Hz
21	WPT-Acc-3	0.013	0.1562 Hz
22	WPT-Acc-4	0.006	0.2188 Hz
23	WPT-Acc-5	0.003	0.2812 Hz
24	WPT-Acc-6	0.003	0.3438 Hz
25	WPT-Acc-7	0.014	0.4062 Hz
26	WPT-Acc-8	0.008	0.4688 Hz
27	WPT-Acc-9	0.004	0.5312 Hz
28	WPT-Acc-10	0.008	0.5938 Hz
29	WPT-Acc-11	0.003	0.6562 Hz
30	WPT-Acc-12	0.002	0.7188 Hz
31	WPT-Acc-13	0.002	0.7812 Hz
32	WPT-Acc-14	0.006	0.8438 Hz
33	WPT-Acc-15	0.005	0.9062 Hz
34	WPT-Acc-16	0.001	0.9688 Hz
35	LSW-HR-Acc-1	0.019	0.75 Hz
36	LSW-HR-Acc-2	0.012	0.375 Hz
37	LSW-HR-Acc-3	0.023	0.1875 Hz
38	**LSW-HR-Acc-4**	0.037	0.0938 Hz
39	LSW-HR-Acc-5	0.018	0.0469 Hz
40	**LSW-HR-Acc-6**	0.048	0.0234 Hz
41	LSW-HR-Acc-7	0.014	0.0117 Hz
42	LSW-HR-Acc-8	0.028	0.0059 Hz
43	**LSW-HR-Acc-9**	0.051	0.0029 Hz
44	LSW-HR-Acc-10	0.018	0.0015 Hz
45	**LSW-HR-Acc-11**	0.060	0.0007 Hz

#	Feature name	Absolute coefficient*	Notes
46	**LSW-HR-Acc-12**	0.040	0.0004 Hz
47	**Duration**	0.465	
48	**Latency**	0.188	
49	**SDRR**	0.113	
50	**Age**	0.120	
51	**Change point binary**	1.808	

*Threshold = 0.03

Table S3. Feature importance and selection criteria. The mean of the absolute coefficients from the Lasso regularised logistic regression model, calculated across the 5-fold cross-validation, was compared to a threshold for feature selection. Selected features are indicated in bold. We also provide the centre frequencies corresponding to the wavelets for WPT-HR-x, WPT-Acc-x, and LSW-HR-Acc-x. WPT-HR: wavelet packet transform of HR; WPT-Acc: wavelet packet transform of Acc; LSW-HR-Acc: local stationary wavelet estimated coherence between HR and Acc.

9. Alternative classifiers

We evaluated the performance of alternative classifiers trained on the same SMOTE-oversampled training set and tested on a consistent test set. The classifiers examined included logistic regression (used in this study), linear discriminant analysis (LDA), quadratic discriminant analysis (QDA), naive Bayes, K-nearest neighbours (KNN), random forest, and neural network. Overall, their performance was comparable, with only minor differences in the balance between precision and recall.

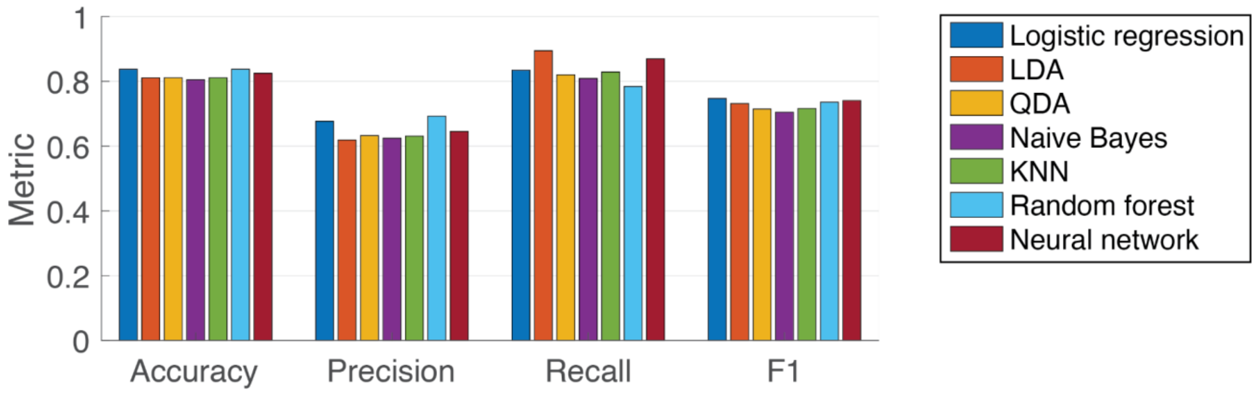

Figure S4. Performance comparison of various classifiers. KNN: 100 neighbours. Neural network: 2 layers, 10 neurons each. Specific values are presented in Table S4.

**Classifier**	**Accuracy**	**Precision**	**Recall**	**F1 score**
Logistic regression	0.8373	0.6765	0.8345	0.7472
LDA	0.8107	0.6187	0.8942	0.7314
QDA	0.8108	0.6326	0.8199	0.7142
Naive Bayes	0.8046	0.6243	0.8091	0.7048
KNN	0.8108	0.6307	0.8288	0.7163
Random forest	0.8373	0.6924	0.7841	0.7354
Neural network	0.8247	0.6454	0.8694	0.7408

Table S4. Performance metrics of alternative classification models. Metrics were calculated based on the test sets.

10. Sensitivity test

To evaluate whether the dataset size was adequate for training the model, we conducted a sensitivity test by training on varying fractions of the dataset and assessing performance on the test set. Results showed that precision, recall, and F1 score stabilized when using more than half of the training set, indicating that our model was trained with a sufficient sample size (Figure S5).

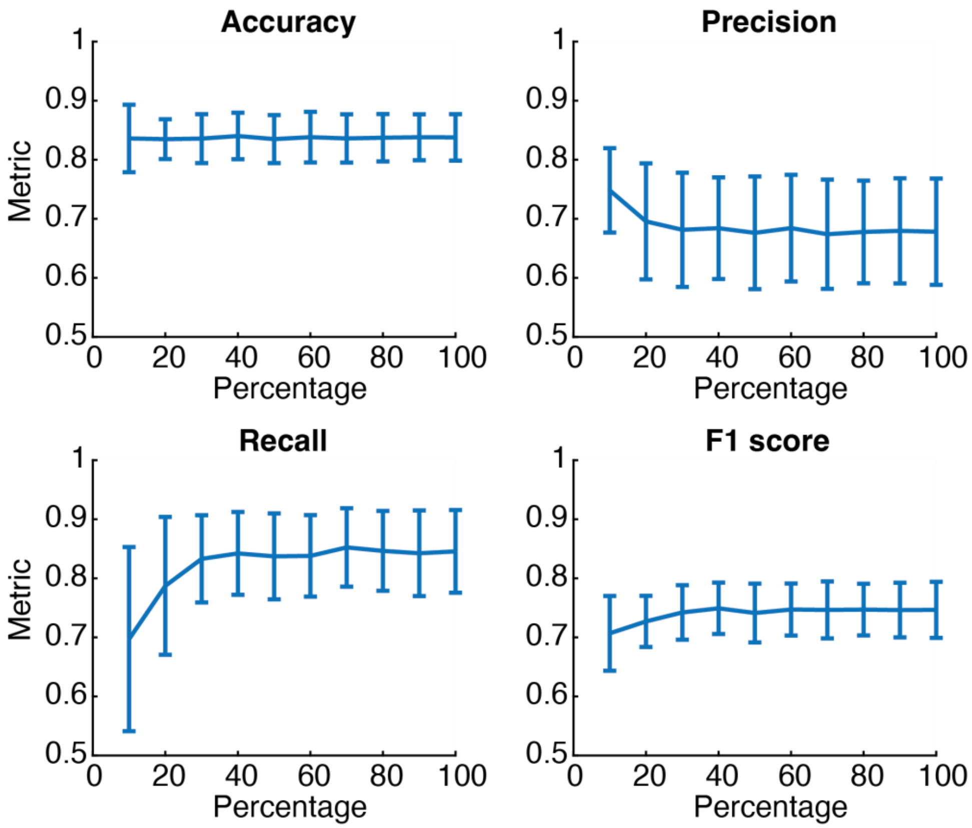

Figure S5. Sensitivity analysis of sample size. The X-axis represents the percentage of the training set used for model training, with evaluations performed on the test set. Lines indicate mean values, and error bars represent standard deviations.

11. Statistics of model application

To evaluate ASAP's effectiveness in detecting natural statistics associated with the attention and inattention states, we extracted the mean visual saliency and mean visual clutter within periods of these two states from each session. We then tested the significance of the following linear model:

$$Measure = \beta_0 + \beta_1 Attention + \beta_2 Source + \beta_3 Age + \beta_4 (Attention \times Source) + \beta_5 (Attention \times Age) + \beta_6 (Source \times Age) + \beta_7 (Attention \times Source \times Age) + \epsilon,$$

where Measure is either Mean Saliency or Mean Clutter. The p-values were compared to a Bonferroni-corrected significance level $\alpha = 0.05/14 = 0.0036$, as there were 14 hypotheses (7 coefficients x 2 dependent variables (saliency and clutter)) to test.

Measure	Fixed effect	Coefficient	P-value*
**Mean Saliency**	**Attention**	0.125	**6.6e-8**
	Source	0.0270	0.23
	Age	0.00111	0.14
	Attention x Source	-0.0761	0.017
	**Attention x Age**	**-0.00426**	**7.7e-5**
	Source x Age	-0.00110	0.30
	Attention x Source x Age	0.00397	0.0086
Mean Clutter	Attention	3.51e-4	0.99
	Source	-0.00555	0.79
	Age	-4.01e-5	0.95
	Attention x Source	0.00627	0.83
	Attention x Age	-3.26e-5	0.97
	Source x Age	2.53e-4	0.80
	Attention x Source x Age	-3.85e-4	0.79

*Significant after Bonferroni correction. $\alpha = 0.05$.

Table S5. Analysis of variance table for model application. The coefficients and p-values were estimated by fitting a linear model.

12. Source of discrepancies between human coder and ASAP labels

The observed variability in performance across sessions prompted us to investigate whether these fluctuations were attributable to the intrinsic characteristics of the dataset or the model's inherent biases. In the first scenario, data with specific characteristics would consistently yield better performance, regardless of whether the labels are from humans or machines. In the second scenario, the model might perform well with certain data types, even though human coders show different consistency.

To test this, we examined whether the level of agreement between human coders (HH) correlated with the agreement between a human coder and the ASAP model (HM). If data characteristics drove the performance variation, we would expect a correlation between HH and HM agreements. For HH agreement, we calculated Cohen's κ between two human coders (see *Inter-rater reliability of human coding*). We calculated Cohen's κ using the same participant sessions (N = 15 subjects) as the HH calculation for HM agreement. The HM agreement was assessed between the machine's predicted attention and Human Coder #1's annotations, which were used for model training.

Our initial correlation analysis revealed a statistically significant positive correlation between HH and HM Cohen's κ values (Pearson's $r = 0.55$, $p = 0.017$, permutation test; Fig. S6a, b), suggesting that specific dataset characteristics might influence performance variability. To investigate potential underlying factors driving this correlation, we examined age, heart rate variability (assessed via SDRR), change point density (number of change points detected per second), and the extent of movement (assessed via the standard deviation of Acc). We tested whether the correlation between HH and HM Cohen's κ was attributable to these factors by calculating partial correlations conditioned on each. Except for SDRR ($p = 0.074$, permutation test), the partial correlations with other factors remained significantly greater than 0 ($p < 0.05$; Fig. S6c). These results suggest that HRV, as measured by SDRR, influenced the uncertainty of attention detection for both humans and ASAP. Specifically, higher HRV was associated with increased inter-rater reliability in identifying periods of attention (Pearson's correlations $r = 0.42$ between SDRR and HH, and $r = 0.55$ between SDRR and HM Cohen's κ). Other possible sources of discrepancies are discussed in the Discussion section but are beyond the scope of the current study.

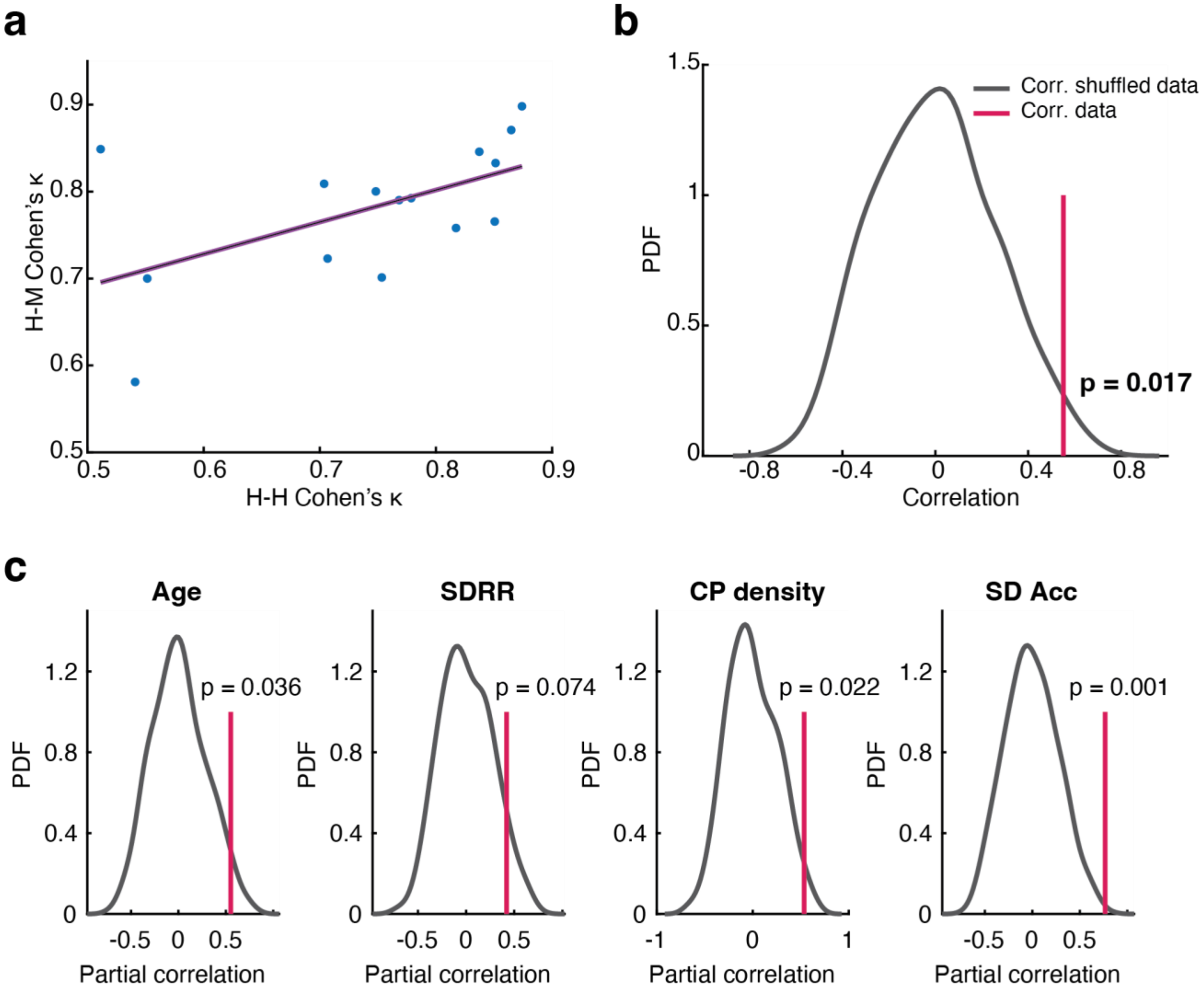

Figure S6. Analysis of the source of disagreement between human coders and ASAP. (a) Scatter plot of H-M vs. H-H Cohen's κ. The line is the linear fit. (b) Distribution of the HH-HM correlation with shuffled data. The red line indicates the data's correlation. (c) Shuffled data establish the null distributions of partial correlations. Red lines indicate the data's partial correlation conditioned on each factor. PDF: probability density function. CP: change point.

Supplemental Material References

1 Kretch, K. S., Franchak, J. M. & Adolph, K. E. Crawling and walking infants see the world differently. *Child Development* **85**, 1503-1518 (2014). https://doi.org/10.1111/cdev.12206

2 Yu, C. & Smith, L. B. Multiple sensory-motor pathways lead to coordinated visual attention. *Cognitive Science* **41**, 5-31 (2017). https://doi.org/10.1111/cogs.12366

3 Hughes, C. & Ensor, R. Executive function and theory of mind: Predictive relations from ages 2 to 4. *Developmental Psychology* **43**, 1447-1459 (2007). https://doi.org/10.1037/0012-1649.43.6.1447

4 Zimmermann, L., Frank, H. E., Subiaul, F. & Barr, R. Applying computational modeling to assess age-, sex-, and strategy-related differences in Spin the Pots, a working memory task for 2- to 4-year-olds. *Developmental Psychobiology* **63**, 42-53 (2021). https://doi.org/10.1002/dev.22016

5 Slone, L. K. *et al.* Gaze in action: Head-mounted eye tracking of children's dynamic visual attention during naturalistic behavior. *JoVE (Journal of Visualized Experiments)*, e58496 (2018).

6 Yu, C. & Smith, L. B. Joint attention without gaze following: Human infants and their parents coordinate visual attention to objects through eye-hand coordination. *PloS one* **8**, e79659 (2013).

7 Mason, H. T. *et al.* A complete pipeline for heart rate extraction from infant ECG. (2024).

8 Makowski, D. *et al.* NeuroKit2: A Python toolbox for neurophysiological signal processing. *Behavior Research Methods* **53**, 1689-1696 (2021). https://doi.org/10.3758/s13428-020-01516-y

9 Bradshaw, J., Fu, X., Yurkovic-Harding, J. & Abney, D. Infant embodied attention in context: Feasibility of home-based head-mounted eye tracking in early infancy. *Developmental Cognitive Neuroscience* **64** (2023). https://doi.org/10.1016/j.dcn.2023.101299

10 Franchak, J. M., Kretch, K. S., Soska, K. C. & Adolph, K. E. Head-mounted eye tracking: A new method to describe infant looking. *Child Development* **82**, 1738-1750 (2011). https://doi.org/10.1111/j.1467-8624.2011.01670.x

11 Kretch, K. S. & Adolph, K. E. The organization of exploratory behaviors in infant locomotor planning. *Developmental Science* **20** (2017). https://doi.org/10.1111/desc.12421

12 Harel, J., Koch, C. & Perona, P. Graph-based visual saliency. *Advances in Neural Information Processing Systems* **19** (2006).

13 Hunter, B. K., Klotz, S., DeBolt, M., Luck, S. & Oakes, L. Evaluation of graph-based visual saliency model using infant fixation data. *Journal of Vision* **23** (2023). https://doi.org/10.1167/jov.23.9.5704

14 Mahdi, A., Su, M., Schlesinger, M. & Qin, J. A comparison study of saliency models for fixation prediction on infants and adults. *IEEE Transactions on Cognitive and Developmental Systems* **10**, 485-498 (2018). https://doi.org/10.1109/tcds.2017.2696439

15 Kadooka, K. & Franchak, J. M. Developmental changes in infants' and children's attention to faces and salient regions vary across and within video stimuli. *Developmental Psychology* **56**, 2065-2079 (2020). https://doi.org/10.1037/dev0001073

16 Rider, A. T., Coutrot, A., Pellicano, E., Dakin, S. C. & Mareschal, I. Semantic content outweighs low-level saliency in determining children's and adults' fixation of movies. *Journal of Experimental Child Psychology* **166**, 293-309 (2018). https://doi.org/10.1016/j.jecp.2017.09.002

17 Sun, L., Francis, D. J., Nagai, Y. & Yoshida, H. Early development of saliency-driven attention through object manipulation. *Acta Psychologica* **243** (2024). https://doi.org/10.1016/j.actpsy.2024.104124

18 Erlikhman, G., Gutentag, S., Blair, C. D. & Caplovitz, G. P. Interactions of flicker and motion. *Vision Research* **155**, 24-34 (2019). https://doi.org/10.1016/j.visres.2018.12.005

19 Rosenholtz, R., Li, Y. & Nakano, L. Measuring visual clutter. *Journal of Vision* **7** (2007). https://doi.org/10.1167/7.2.17

20 Haynes, K., Eckley, I. A. & Fearnhead, P. Computationally efficient changepoint detection for a range of penalties. *Journal of Computational and Graphical Statistics* **26**, 134-143 (2017). https://doi.org/10.1080/10618600.2015.1116445

21 Fryzlewicz, P. Wild binary segmentation for multiple change-point detection. *The Annals of Statistics* **42** (2014). https://doi.org/10.1214/14-aos1245

22 Fryzlewicz, P. Detecting possibly frequent change-points: Wild Binary Segmentation 2 and steepest-drop model selection. *Journal of the Korean Statistical Society* **49**, 1027-1070 (2020). https://doi.org/10.1007/s42952-020-00060-x

23 Donoho, D. L. & Johnstone, I. M. Ideal spatial adaptation by wavelet shrinkage. *Biometrika* **81**, 425-455 (1994). https://doi.org/10.1093/biomet/81.3.425

24 Truong, C., Oudre, L. & Vayatis, N. Selective review of offline change point detection methods. *Signal Processing* **167** (2020). https://doi.org/10.1016/j.sigpro.2019.107299

25 Killick, R., Fearnhead, P. & Eckley, I. A. Optimal detection of changepoints with a linear computational cost. *Journal of the American Statistical Association* **107**, 1590-1598 (2012). https://doi.org/10.1080/01621459.2012.737745

26 Killick, R. & Eckley, I. A. changepoint: An R package for changepoint analysis. *Journal of Statistical Software* **58** (2014). https://doi.org/10.18637/jss.v058.i03

27 Akaike, H. A new look at the statistical model identification. *IEEE Transactions on Automatic Control* **19**, 716-723 (1974). https://doi.org/10.1109/tac.1974.1100705

28 Schwarz, G. Estimating the dimension of a model. *The annals of statistics*, 461-464 (1978).

29 Zhang, N. R. & Siegmund, D. O. A modified Bayes information criterion with applications to the analysis of comparative genomic hybridization data. *Biometrics* **63**, 22-32 (2007). https://doi.org/10.1111/j.1541-0420.2006.00662.x

30 Hannan, E. J. & Quinn, B. G. The determination of the order of an autoregression. *Journal of the Royal Statistical Society Series B: Statistical Methodology* **41**, 190-195 (1979). https://doi.org/10.1111/j.2517-6161.1979.tb01072.x

31 Baranowski, R., Chen, Y. & Fryzlewicz, P. Narrowest-over-threshold detection of multiple change points and change-point-like features. *Journal of the Royal Statistical Society Series B: Statistical Methodology* **81**, 649-672 (2019). https://doi.org/10.1111/rssb.12322

32 Cho, H. & Kirch, C. Two-stage data segmentation permitting multiscale change points, heavy tails and dependence. *Annals of the Institute of Statistical Mathematics* **74**, 653-684 (2021). https://doi.org/10.1007/s10463-021-00811-5

33 Anastasiou, A. & Fryzlewicz, P. Detecting multiple generalized change-points by isolating single ones. *Metrika* **85**, 141-174 (2021). https://doi.org/10.1007/s00184-021-00821-6

34 Fryzlewicz, P. Tail-greedy bottom-up data decompositions and fast multiple change-point detection. *The Annals of Statistics* **46** (2018). https://doi.org/10.1214/17-aos1662

35 Wood, S. N. *Generalized additive models: an introduction with R*. (chapman and hall/CRC, 2017).
